# Endothelial SIRT3 regulates myofibroblast metabolic shifts in diabetic kidneys

**DOI:** 10.1016/j.isci.2021.102390

**Published:** 2021-04-06

**Authors:** Swayam Prakash Srivastava, Jinpeng Li, Yuta Takagaki, Munehiro Kitada, Julie E. Goodwin, Keizo Kanasaki, Daisuke Koya

**Affiliations:** 1 Department of Diabetology & Endocrinology, Kanazawa Medical University, Uchinada, Ishikawa 920-0293, Japan; 2 Department of Anticipatory Molecular Food Science and Technology, Medical Research Institute, Kanazawa Medical University, Uchinada, Ishikawa 920-0293, Japan; 3 Internal Medicine 1, Shimane University, Faculty of Medicine, Izumo, Shimane 693-8501, Japan; 4 Department of Pediatrics (Nephrology) Yale University School of Medicine, New Haven, CT 06520, USA; 5 Vascular Biology and Therapeutics Program, Yale University School of Medicine New Haven, CT 06520, USA

**Keywords:** Biological Sciences, Cell Biology, Functional Aspects of Cell Biology

## Abstract

Defects in endothelial cells cause deterioration in kidney function and structure. Here, we found that endothelial SIRT3 regulates metabolic reprogramming and fibrogenesis in the kidneys of diabetic mice. By analyzing, gain of function of the SIRT3 gene by overexpression in a fibrotic mouse strain conferred disease resistance against diabetic kidney fibrosis, whereas its loss of function in endothelial cells exacerbated the levels of diabetic kidney fibrosis. Regulation of endothelial cell SIRT3 on fibrogenic processes was due to tight control over the defective central metabolism and linked activation of endothelial-to-mesenchymal transition (EndMT). SIRT3 deficiency in endothelial cells stimulated the TGFβ/Smad3-dependent mesenchymal transformations in renal tubular epithelial cells. These data demonstrate that SIRT3 regulates defective metabolism and EndMT-mediated activation of the fibrogenic pathways in the diabetic kidneys. Together, our findings show that endothelial SIRT3 is a fundamental regulator of defective metabolism regulating health and disease processes in the kidney.

## Highlights

• Endothelial SIRT3 protects against renal fibrosis in diabetes
• Endothelial SIRT3 regulates mesenchymal metabolic shifts
• Amelioration of endothelial SIRT3 could be a potential therapeutic approach
• Targeting defective metabolism offers new therapeutics against kidney diseases

## Graphical abstract

**Figure:**
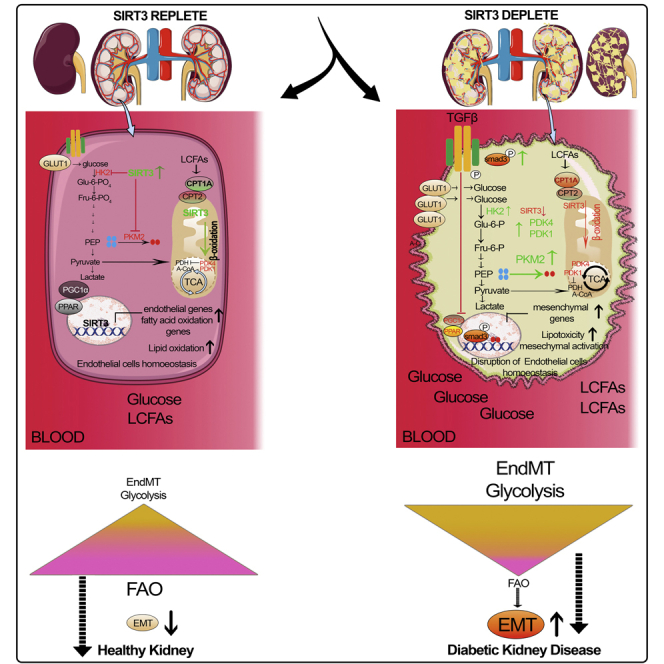

## Introduction

Of over 400 million people with diabetes, about one-third will develop diabetic kidney disease (DKD), a leading cause of end-stage renal disease that causes more than 950,000 deaths each year globally (Badal and Danesh, 2014; Cooper and Warren, 2019; Reidy et al., 2014). Over the last two decades, no new drugs have been approved to specifically prevent DKD or to improve kidney functions (Breyer and Susztak, 2016). This lack of progress likely stems from poor understanding of the mechanism of kidney dysfunction, and this knowledge gap contributes to the suboptimal treatment options available for these patients. Improved understanding of the mechanism of pathogenesis of DKD is urgently needed to catalyze the development of novel therapeutics that can be targeted to the early stages of these diseases, before kidney damage becomes irreversible.

Although kidney fibrosis is identical manifestation in all progressive form of chronic kidney disease and DKD, however, it is caused by excess deposition of extracellular matrix leading to renal function deterioration, and kidney injury (LeBleu et al., 2013; Srivastava et al., 2019; Zeisberg et al., 2003). Fibroblasts play a crucial role in kidney fibrosis, but the origin of fibroblasts is still obscure (Grande and Lopez-Novoa, 2009; LeBleu et al., 2013; Srivastava et al., 2013; Zeisberg and Neilson, 2010). There are six well-reported sources of matrix-producing myofibroblasts: (1) activated residential fibroblasts, (2) differentiated pericytes, (3) recruited circulating fibrocytes, (4) from mesenchymal cells derived from M2 phenotype macrophages via macrophage-to-mesenchymal transition, (5) from mesenchymal cells derived from tubular epithelial cells via epithelial-to-mesenchymal transition (EMT), and (6) from mesenchymal cells transformed from endothelial cells (ECs) via endothelial-to-mesenchymal transition (EndMT) (Grande and Lopez-Novoa, 2009; Grande et al., 2015; LeBleu et al., 2013; Srivastava et al., 2019; Wang et al., 2017). Among these diverse origins of matrix-producing fibroblasts, EndMT is an important source of myofibroblasts in several organs, including in the kidney (Medici, 2016; Srivastava and Goodwin, 2020; Srivastava et al., 2019). EndMT is characterized by the loss of endothelial markers, including cluster of differentiation 31 (CD31), and acquisition of the expression of mesenchymal proteins including α-smooth muscle actin (αSMA), fibronectin, and SM22α (Srivastava et al., 2019). The complete conversion from ECs into mesenchymal cell types is not essential, even the intermediate cell phenotypes are sufficient to induce the fibrogenic programs (Kizu et al., 2009; LeBleu et al., 2013; Zeisberg et al., 2007).

ECs are key players in the formation of new blood vessels both in health and life-threatening diseases (Eelen et al., 2015, 2018). 6-Phosphofructo-2-kinase/fructose-2,6-bisphosphatase-3 (PFKFB3)-driven glycolysis regulates the EC metabolism and vessel sprouting, whereas carnitine palmitoyltransferase 1a (CPT1a)-mediated fatty acid oxidation regulates the proliferation of ECs in the stalk of the sprout (Cantelmo et al., 2016; Cruys et al., 2016; De Bock et al., 2013; Schoors et al., 2014, 2015). To maintain vascular homeostasis, ECs use metabolites for epigenetic regulation of EC sub-type differentiation and maintain cross talk through metabolite release with other cell types (Eelen et al., 2018; Schoors et al., 2015). Disruption of metabolic homeostasis in ECs contributes to disease phenotypes (Theodorou and Boon, 2018; Zhou et al., 2019). Importantly, EndMT causes alteration of EC structure and metabolism, which is an area of active investigation (Chen et al., 2012; Lovisa and Kalluri, 2018; Xiong et al., 2018). The mesenchymal cells derived from EndMT reprogram their metabolism and depend on glycolytic metabolites for nucleic acid, amino acids, glycoproteins, and lipid synthesis (Eelen et al., 2018; Lovisa and Kalluri, 2018; Theodorou and Boon, 2018; Xiong et al., 2018). Researchers have examined the contribution of EndMT to renal fibrosis in several mouse models of chronic kidney disease (Grande and Lopez-Novoa, 2009; LeBleu et al., 2013; Liu, 2011; Medici and Kalluri, 2012; Srivastava et al., 2014; Zeisberg et al., 2008; Zeisberg and Neilson, 2010). Zeisberg et al. performed seminal experiment and reported that approximately 30%–50% of fibroblasts co-expressed the endothelial marker CD31 and markers of fibroblasts and myofibroblasts such as fibroblast-specific protein-1 (FSP-1) and αSMA in the kidneys of mice that had unilateral ureteral obstructive nephropathy (Zeisberg et al., 2008). EndMT contributes to the accumulation of activated fibroblasts; thus, targeting EndMT might have therapeutic potential against renal fibrosis (LeBleu et al., 2013; Li et al., 2020a; Liu, 2011; Medici and Kalluri, 2012; Srivastava et al., 2020a; Zeisberg et al., 2008).

A correlation between mitochondrial damage, inflammation, and renal fibrosis has been demonstrated suggesting that mitochondrial integrity and mitochondrial metabolism are critical for kidney cells' homeostasis (Chung et al., 2019). Mitochondrial sirtuins play a key role in the regulation of mitochondrial integrity and metabolism, and during recent years the involvement of mitochondrial sirtuins is gaining momentum in kidney research (Hershberger et al., 2017; Morigi et al., 2015; Perico et al., 2016; Srivastava et al., 2020c). Among mitochondrial sirtuins, SIRT3 is a major deacetylase that targets several diverse enzymes involved in central metabolism, resulting in the activation of many oxidative pathways (Yin and Cadenas, 2015). SIRT3 blocks the characteristics of organ fibrosis by regulating TGF-β/smad signaling (Chen et al., 2015; Sosulski et al., 2017; Sundaresan et al., 2015). SIRT3 regulates the abnormal glucose metabolism via tight control over PKM2 tetramer-to-dimer interconversion and HIF1α accumulation in the diabetic kidneys (Srivastava et al., 2018). SIRT3-depleted tubular epithelial cells are highly dependent on reprogrammed defective metabolism and are associated with higher mesenchymal activation in the diabetic kidneys (Srivastava et al., 2018, 2020b).

In the present study, we aimed to understand the contribution of EC SIRT3 in the regulation of metabolic reprograming and fibrogenic processes in the kidneys. Therefore the development of suitable animal models for studying the functional and physiological implication of EC SIRT3 is critical to understand the pathogenesis of DKD. To begin to understand how EC SIRT3 may be regulating renal fibrosis in diabetes, we developed the following two unique novel mouse models: (1) overexpression mouse model in fibrotic background and (2) endothelial-specific deletion of SIRT3 gene in less-fibrotic mouse background.

Our results indicate a key role of EC SIRT3 in the regulation the metabolic reprogramming and linked activation of EndMT processes, which contributes to fibrogenic phenotype in the kidneys of diabetic mice.

## Results

### SIRT3 deficiency in endothelial cells is a critical fibrogenic phenotype in the kidneys of diabetic mice

**Figure 1:**
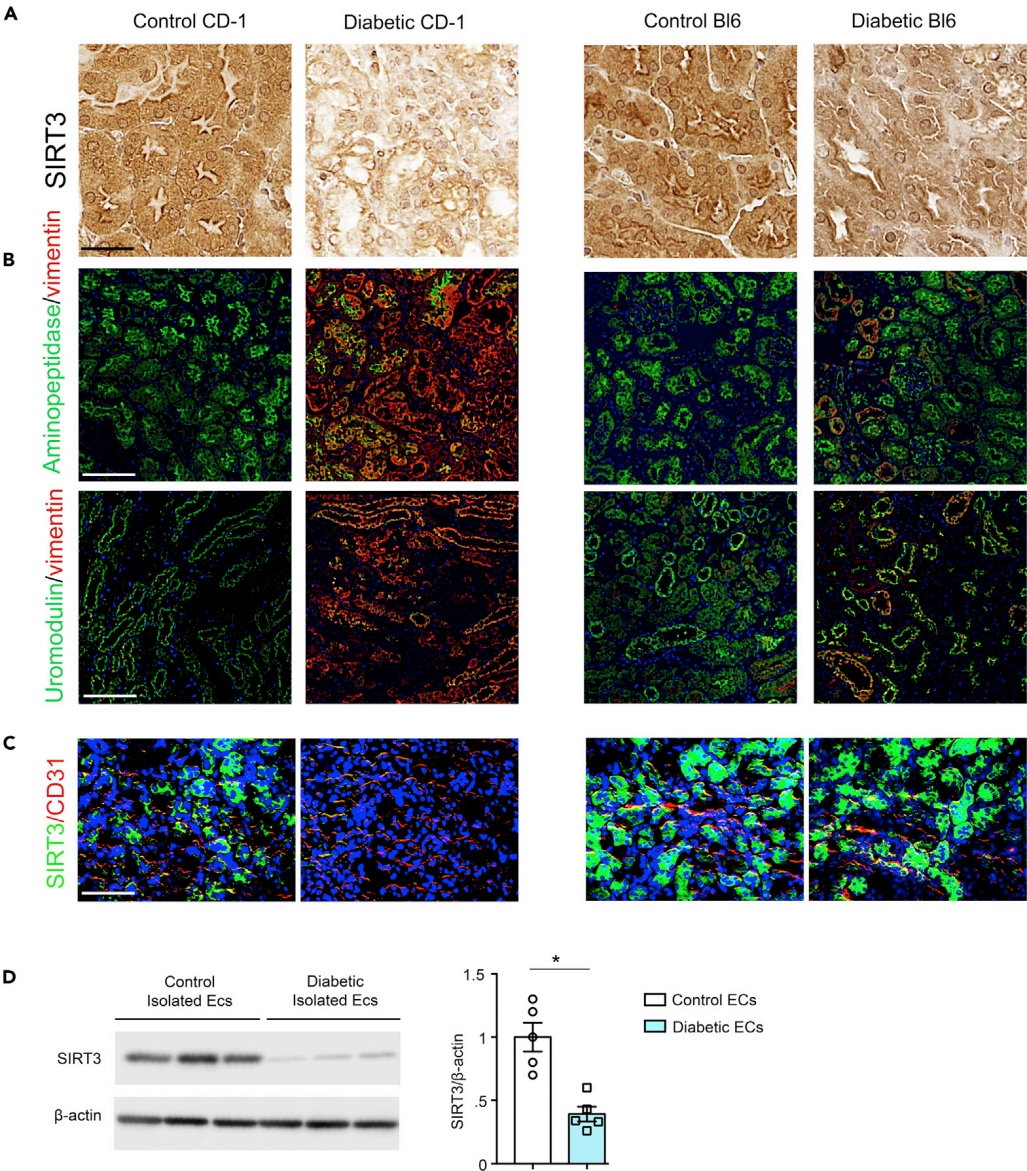
Diabetic kidney disease is associated with suppression of endothelial SIRT3 protein (A) Immunohistochemical analysis of the kidneys of control and diabetic CD-1 and C57BL/6 mice. Representative pictures are shown. 40× images are shown. N = 7 per group. Scale bar, 50 μm. (B) Immunofluorescence analysis for aminopeptidase A/vimentin and uromodulin/vimentin in the kidneys of control and diabetic CD-1 and C57BL/6 mice. Representative pictures are shown. N = 7 per group. Scale bar, 50 μm. (C) Immunofluorescence analysis of the kidneys of control and diabetic CD-1 and C57BL/6 mice. FITC-labeled SIRT3, rhodamine-labeled CD31, and DAPI blue. Scale bar, 50 μm. Representative pictures are shown. 40× images are shown. N = 7 for CD1 mice, whereas N = 5 for C57BL/6 mice. Data in the graph are shown as mean ± SEM. (D) Western blot analysis of SIRT3 protein in isolated endothelial cells from the kidneys of control and diabetic CD-1 mice. Densitometry analysis was normalized by β-actin. N = 5 were analyzed in each group. Representative blots are shown. Data in the graph are shown as mean ± SEM. Student's t test was used for the analysis of statistical significance. ∗p < 0.05.

Streptozotocin (STZ)-induced diabetic CD-1 is the established mouse model to study DKD (Kanasaki et al., 2014; Nagai et al., 2014; Nitta et al., 2016; Shi et al., 2015; Srivastava et al., 2016). In mice, the kidney fibrosis phenotype is largely dependent on the strain specificity (Srivastava et al., 2016; Sugimoto et al., 2007). The STZ-induced diabetic CD-1 mice and diabetic C57BL/6 mice experienced similar levels of blood glucose; however, the kidneys of diabetic CD-1 mice have been shown to display higher rate of EndMT and massive fibrosis when compared with the kidneys of diabetic C57BL/6 mice (Kanasaki et al., 2014; Srivastava et al., 2018). Here, we confirmed the dose-dependent effect of STZ in the development of renal fibrosis in the CD-1 and C57BL/6 mouse strains and found that the kidneys of diabetic CD-1 mice experienced higher fibrosis when compared with diabetic C57BL/6 mice (Figures S1A and S1B). Therefore diabetic CD-1 mouse is known as the fibrotic strain; however, the diabetic C57BL/6 mouse is considered as a less-fibrotic strain (Srivastava et al., 2018; Sugimoto et al., 2007). The kidneys of diabetic CD-1 mice displayed complete suppression of SIRT3 protein when compared with the non-diabetic control, whereas diabetic C57BL/6 did not (Figure 1A). Multiplex immunohistochemical analysis of vimentin/aminopeptidase A (a marker of proximal tubule) or vimentin/uromodulin (a marker of distal tubule) showed higher level of vimentin in the proximal and distal tubules in the kidneys of diabetic CD-1 mice when compared with non-diabetic control, and such effects were not prominent in the kidneys of diabetic C57BL/6 mice (Figure 1B). Furthermore, we found that CD31-positive cells in diabetic CD-1 mouse kidneys displayed significant suppression of SIRT3, whereas the kidneys of diabetic C57BL/6 mice did not reveal a remarkable difference in SIRT3 protein levels (Figure 1C). In addition, we found that the ECs isolated from the kidneys of diabetic CD-1 mice showed significant suppression of SIRT3 and CD31 protein levels (Figure 1D).

### SIRT3 overexpression in fibrogenic phenotype protects from diabetes-associated renal fibrosis

**Figure 2:**
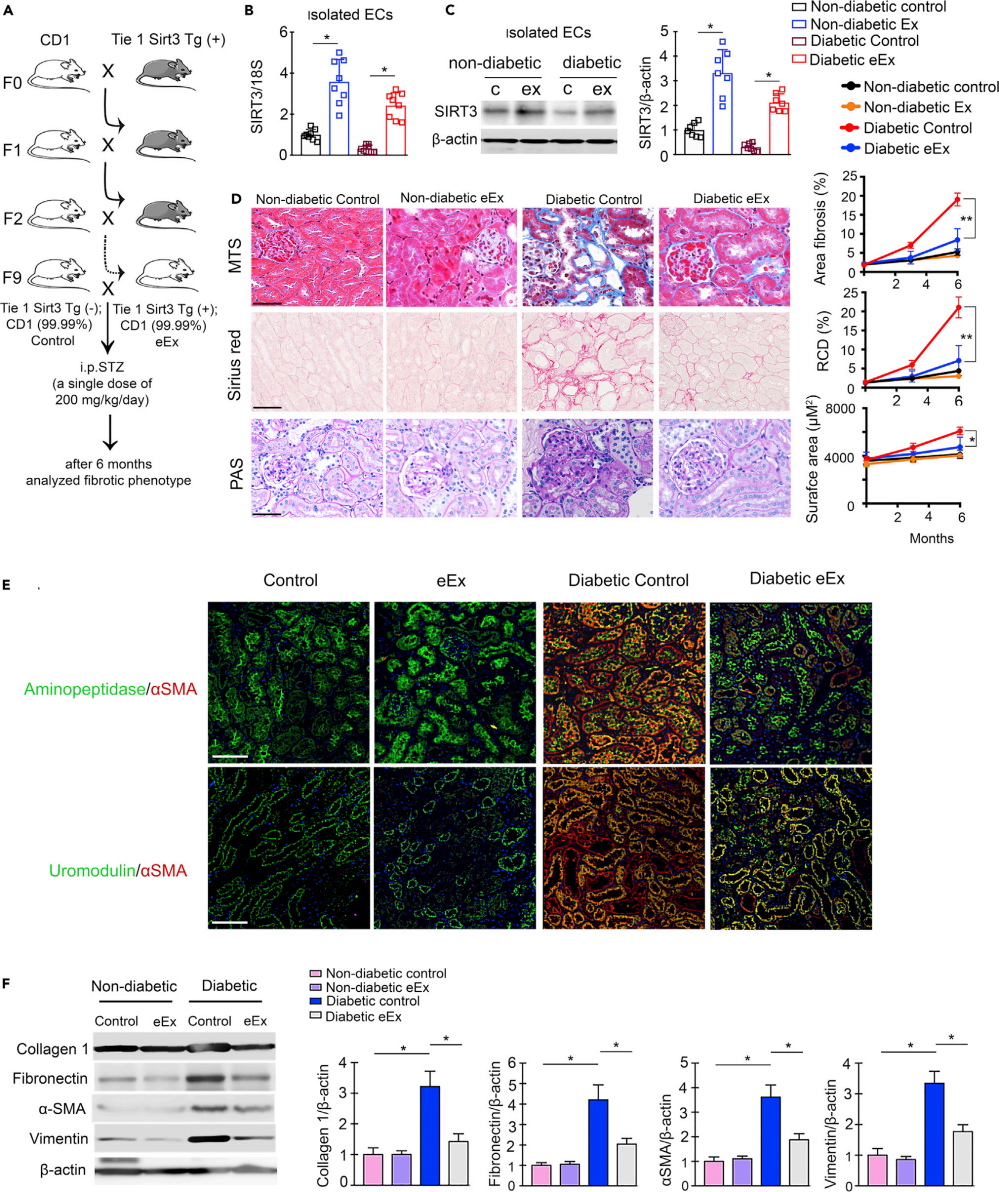
Overexpression of endothelial SIRT3 protects against fibrosis in the kidney of diabetic mice (A) Schematic chart of backcrossing of endothelial-specific Sirt3tg (Tie 1 Sirt3 tg+) with CD-1 mice. After the ninth generation, 99.99% of the genetic background of CD-1 mice was transferred. A single dose of STZ (200 mg/kg/day i.p.) was injected in the control (Tie 1 Sirt3 tg−; CD-1) and eEx (Tie 1 Sirt3 tg+; CD-1) mice to induce fibrosis. (B) SIRT3 mRNA expression was analyzed by qPCR in the isolated endothelial cells of eEX and control littermates. 18S was used as internal control. N = 8 per group. (C) Western blot analysis of SIRT3 protein in isolated endothelial cells from the kidney of control and eEx mice. N = 7 per group. Representative blots are shown. Densitometry calculation was normalized to β-actin. (D) Masson trichrome, Sirius red, and PAS staining in the kidney of non-diabetic and diabetic eEx and control littermates. Representative images are shown. Area of fibrosis (%), relative collagen deposition (RCD %), and surface area (μm^2^) were measured using the ImageJ program. N = 7 per group. Scale bar, 50 μm. 40× images in the MTS and PAS, whereas 30× images in Sirius red. Data in the graph are shown as mean ± SEM. (E) Immunofluorescence analysis for aminopeptidase A/αSMA and uromodulin/αSMA in the kidneys of non-diabetic and diabetic control and eEx mice. Representative images are shown. Scale bar, 50 mm in each panel. N = 7 per group. (F) Western blot analysis of collagen I, fibronectin, α-SMA, and vimentin in the kidney of non-diabetic and diabetic control and eEx mice. N = 5 per group. Representative blots are shown. Densitometry calculation was normalized to β-actin. Data in the graph are shown as mean ± SEM. One-way ANOVA Tukey post hoc test was used for the analysis of statistical significance. ∗p < 0.05.

Our general approach was to define how endogenous SIRT3 in the EC was associated to repress the fibrogenic characteristics in diabetic kidney? To answer, we bred SIRT3 overexpression mouse in the fibrogenic mouse background (CD-1 mouse background). We backcrossed Tie1-Sirt3 tg mice (C57BL/6 background that displayed expressed levels of SIRT3 protein in the ECs) with CD-1 mice. The purpose of backcross breeding was to transfer the Sirt3 Tg gene (which is in less-fibrotic C57BL/6 background) to the ECs of CD-1 mouse (fibrotic phenotype). The schematic diagram depicts the backcross scheme between CD-1 and Sirt3 tg mice (Figure 2A). At the ninth generation, SIRT3 mRNA/protein level was significantly upregulated in the ECs isolated from the kidneys of SIRT3 overexpressing Sirt3 Tg (+); CD-1 mice (from here denoted eEx) when compared with Sirt3 Tg−; CD-1 (control) mice (Figures 2B and 2C). We injected a single higher dose of STZ (200 mg/kg/day intraperitoneaally [i.p.]) in the eEx and littermate control. At the time of sacrifice, non-diabetic eEX and non-diabetic controls had similar body weight, blood glucose, kidney weight, albumin-to-creatinine ratio (ACR), blood pressure, liver weight, and heart weight. However, diabetic eEx had relatively lower kidney weight and significantly suppressed ACR levels when compared with diabetic controls (Figure S2). We did not observe any remarkable differences in the body weight, blood glucose, blood pressure, liver weight, or heart weight in the diabetic eEx when compared with the diabetic controls (Figure S2). We observed minor fibrotic alterations between non-diabetic control and non-diabetic eEx; however, diabetic eEx exhibited significantly lower levels of relative area fibrosis, relative collagen deposition (RCD), and glomerular surface area when compared with diabetic controls (Figures 2D and S3). The diabetic kidneys of female mice displayed significantly less fibrosis when compared with the diabetic kidneys of male mice; moreover, the kidneys of diabetic female eEX mice displayed suppressed level of fibrosis when compared with the kidneys of diabetic female control mice (Figures 2D and S4). Multiplex immunohistochemical data of aminopeptidase/α-SMA and uromodulin/α-SMA revealed a significant reduction in α-SMA level in the proximal tubules and in distal tubules in the kidneys of diabetic eEx (Figure 2E). The kidneys of diabetic eEx mice had suppressed protein level of collagen I, fibronectin, vimentin, and α-SMA when compared with the kidneys of diabetic control (Figure 2F).

### Loss of endothelial SIRT3 worsens diabetes-associated fibrosis in kidney

**Figure 3:**
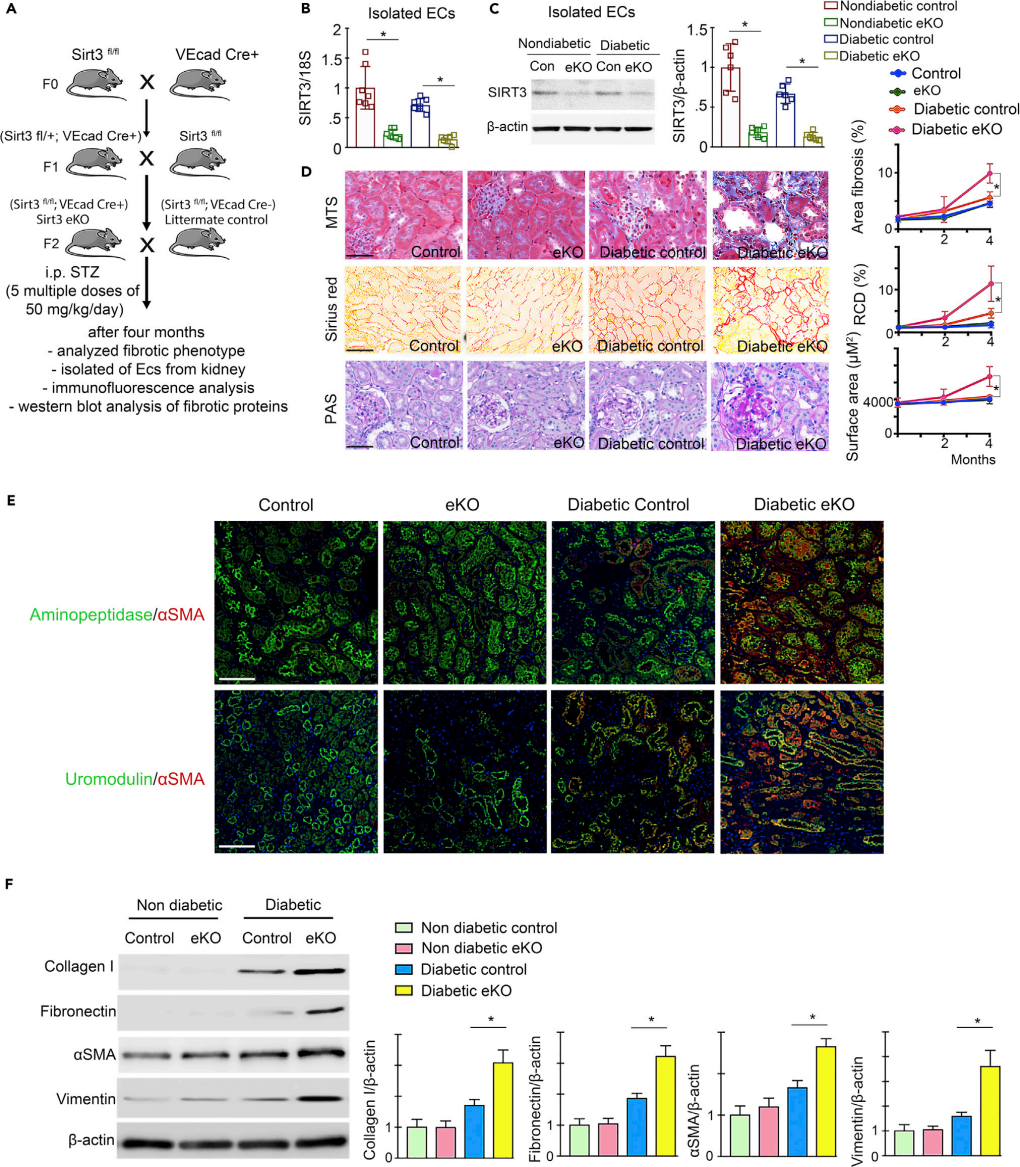
Loss of endothelial SIRT3 worsens renal fibrosis in the mouse model of diabetic kidney disease (A) Schematic chart showing the generation of endothelial-specific Sirt3 knockout mice. Five multiple low doses of STZ (50 mg/kg/day i.p.) were injected in the control (Sirt3 ^fl/fl^; VeCad Cre−) and eKO (Sirt3 ^fl/fl^; VeCad Cre+) mice to induce fibrosis. (B) SIRT3 mRNA expression level was analyzed by qPCR in the isolated endothelial cells of eKO and control littermates. 18S was used as internal control. N = 7 per group. (C) Western blot analysis of SIRT3 protein in isolated endothelial cells from the kidneys of control and eKO mice. N = 6 per group. Representative blots are shown. (D) Masson trichrome, Sirius red, and PAS staining in the kidneys of non-diabetic and diabetic control littermates and eKO mice. Representative images are shown. Area of fibrosis (%), relative collagen deposition (RCD %), and surface area were measured using the ImageJ program. N = 7 per group. Data in the graph are shown as mean ± SEM. Scale bar: 50 μm in MTS and PAS panel and 70 μm in Sirius red. 40× images in the MTS and PAS and 30× images in the Sirius red panel. (E) Immunofluorescence analysis for aminopeptidase A/αSMA and uromodulin/αSMA in the kidneys of non-diabetic and diabetic control littermates and eKO mice. Representative images are shown. Scale bar, 50 mm in each panel. N = 7 per group. (F) Western blot analysis of collagen I, fibronectin, α-SMA, and vimentin in the kidney of non-diabetic and diabetic control littermates and eKO mice. N = 6 per group. Representative blots are shown. Densitometry calculation was normalized to β-actin. Data in the graph are shown as mean ± SEM. One-way ANOVA Tukey post hoc test was used for the analysis of statistical significance. ∗p < 0.05.

To study the loss of function of SIRT3, we deleted the *Sirt3* gene in ECs. Crossing of VE cadherin− Cre mice with mice having floxed alleles of SIRT3 resulted in the excision of the *Sirt3* gene (SIRT3 fl/fl; VE cadherin Cre+; from here denoted eKO), leading to the absence of SIRT3 protein expression in ECs of the kidney when compared with littermate controls (SIRT3 fl/fl; VE cadherin Cre−), which exhibit no recombinase activity. The schematic diagram depicts the generation of eKO mice (Figure 3A). SIRT3 mRNA/protein levels were significantly diminished in the ECs isolated from the kidneys of eKO mice (Figures 3B and 3C). At the age of 10 weeks, we injected five consecutive multiple low doses of STZ (50 mg/kg/day i.p.) and after 4 months analyzed the level of fibrosis in the eKO and littermate control. At the time of sacrifice, non-diabetic eKO and non-diabetic littermate controls had no remarkable change in body weight, blood glucose, kidney weight, ACR, blood pressure, liver weight, and heart weight; however, diabetic eKO had relatively higher kidney weight and significantly higher ACR when compared with diabetic littermate controls (Figure S5). We did not observe a remarkable difference in the body weight, blood glucose, blood pressure, liver weight, or heart weight in the diabetic eKO when compared with diabetic littermate controls (Figure S5). Minor fibrotic alterations between non-diabetic controls and non-diabetic eKO were observed; however, diabetic eKO exhibited higher relative area of fibrosis, RCD, and severe glomerulosclerosis when compared with diabetic littermate controls (Figures 3D and S6). The diabetic kidneys of female mice displayed significantly less fibrosis when compared with the diabetic kidneys of male mice; moreover, the kidneys of diabetic female eKO mice displayed higher level of fibrosis when compared with the kidneys of diabetic female control mice (Figures 3D and S7). Multiplex immunohistochemical data of aminopeptidase/α-SMA and uromodulin/α-SMA revealed a significant higher α-SMA level in the proximal tubules and in distal tubules in the kidneys of diabetic eKO when compared with diabetic control (Figure 3E). The kidneys of diabetic eKO mice had induced protein level of collagen I, fibronectin, vimentin, and α-SMA when compared with the kidneys of diabetic control (Figure 3F).

### SIRT3 regulates endothelial-to-mesenchymal transitions in the kidneys

**Figure 4:**
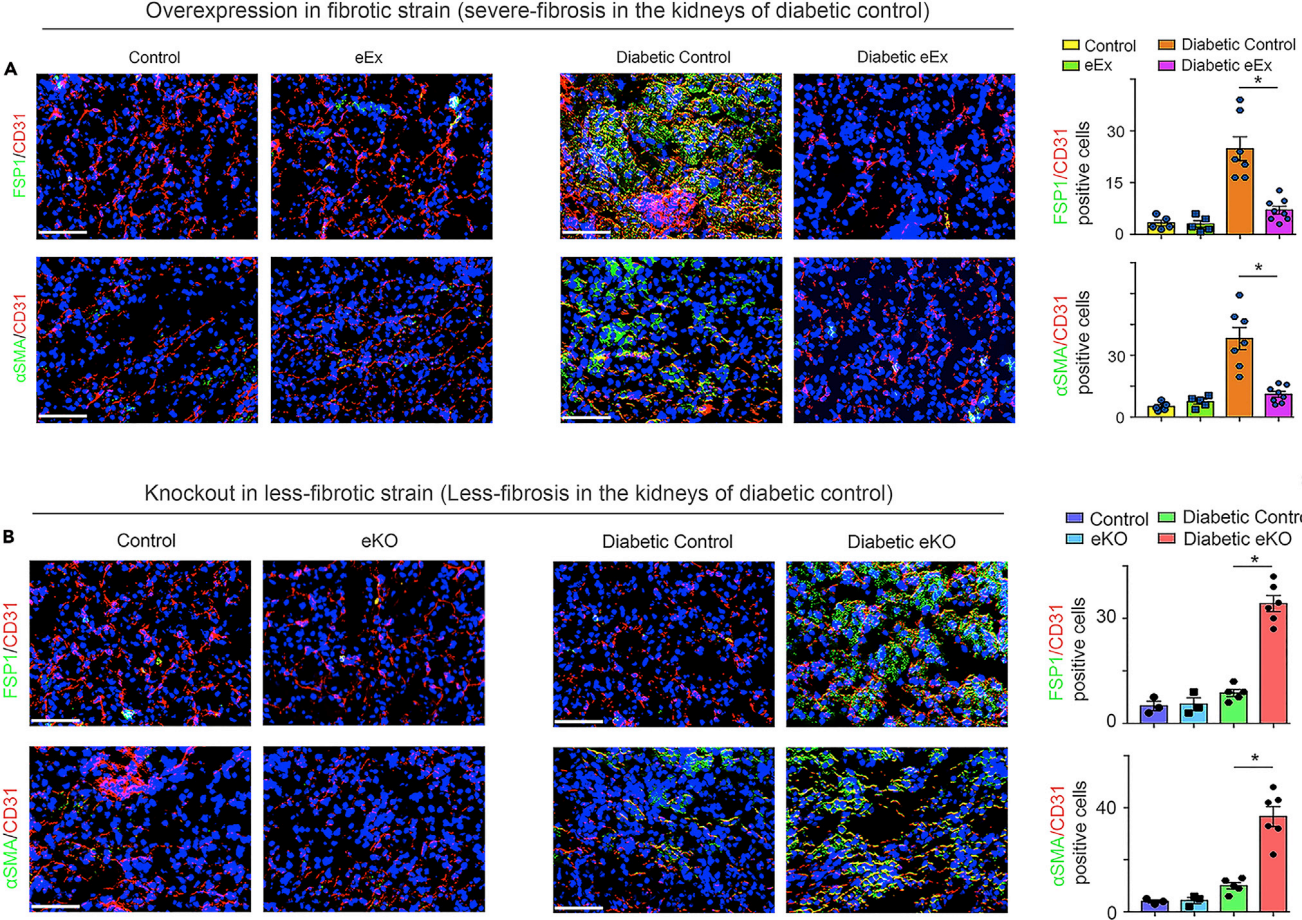
SIRT3 regulates the endothelial-to-mesenchymal transition in the kidney (A) Immunofluorescence analysis was performed in the kidneys of non-diabetic and diabetic control littermates and eEx mice by fluorescence microscopy. FSP-1 and α-SMA protein levels were analyzed in CD31-positive cells. Merged and representative pictures are shown. N = 5 non-diabetic group, N = 7 diabetic control group, N = 8 diabetic eEx group. (B) Immunofluorescence analysis was performed in the kidneys of non-diabetic and diabetic control and eKO mice by fluorescence microscopy. FSP-1 and α-SMA protein levels were analyzed in CD31-positive cells. Merged and representative pictures are shown. N = 3 non-diabetic group, N = 5 diabetic control group, N = 6 diabetic eKO group. Scale bar, 50 mm in each panel. 40× images are shown. Data in the graph are shown as mean ± SEM. One-way ANOVA Tukey post hoc test was used for the analysis of statistical significance. ∗p < 0.05.

The kidneys of diabetic eEX exhibited suppressed levels of FSP-1 and αSMA in CD31-positive cells when compared with diabetic controls (severe fibrosis in kidneys), whereas the kidneys of diabetic eKO displayed significantly higher levels of FSP-1, αSMA, and TGFβR1 in CD31-positive cells when compared with diabetic littermate controls (less fibrosis in kidneys) (Figures 4A and 4B). However, there were no remarkable alterations in the levels of FSP-1 and αSMA in the kidneys of non-diabetic mice (Figures 4A and 4B).

### SIRT3 regulates metabolic reprogramming in the endothelial cells-derived myofibroblasts in the diabetic kidneys

**Figure 5:**
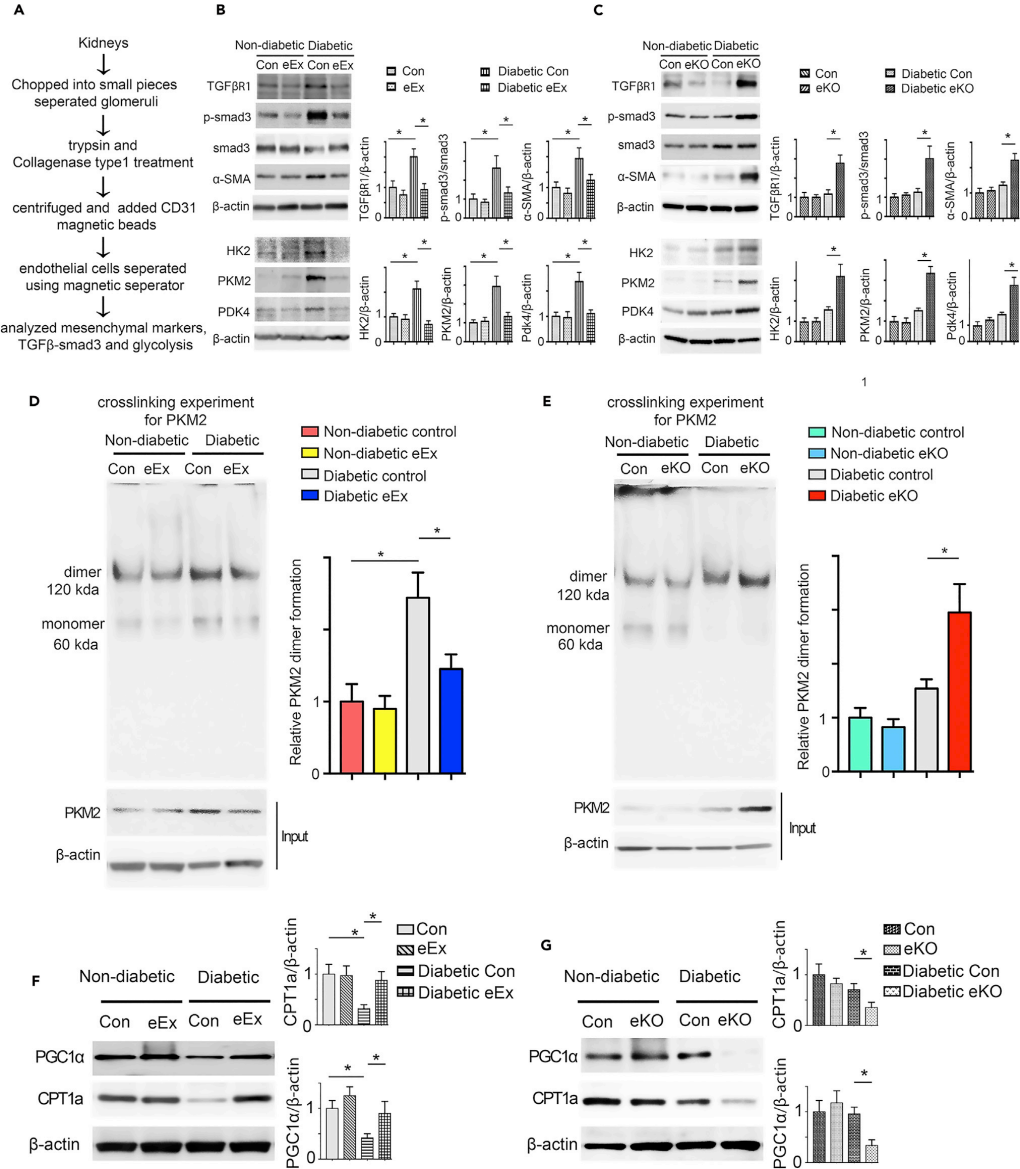
SIRT3 regulates metabolic reprogramming in the endothelial cells-derived fibroblasts in kidney (A) Schematic diagram showing the isolation of endothelial cells from the non-diabetic and diabetic mice. (B) Western blot analysis of TGFβR1, smad3 phosphorylation, total smad3, α-SMA, HK2, PKM2, and PDK4 in the lysates of isolated endothelial cells from non-diabetic and diabetic kidneys of control littermates and eEx mice. Representative blots are shown. Densitometry calculations were normalized to β-actin. N = 6 were analyzed in each group. (C) Western blot analysis of TGFβR1, smad3 phosphorylation, total smad3, α-SMA, HK2, PKM2, and PDK4 in the lysates of isolated endothelial cells from non-diabetic and diabetic kidneys of control and eKO mice. Representative blots are shown. Densitometry calculations were normalized to β-actin. N = 5 for non-diabetic group, N = 6 for diabetic group. (D) Glutaraldehyde chemical cross-linking experiment for PKM2 was performed in the isolated endothelial cells from the non-diabetic and diabetic kidneys of control and eEx mice. The representative blot from five blots is shown. N = 5 per group. (E) Glutaraldehyde chemical cross-linking experiment for PKM2 was performed in the isolated endothelial cells from the non-diabetic and diabetic kidneys of control and eKO mice. The representative blot from five blots is shown. N = 5 per group. (F) Western blot analysis of CPT1a and PGC1α in the lysates of isolated endothelial cells from the non-diabetic and diabetic kidneys of control and eEx mice. Representative blots are shown. Densitometry calculations were normalized to β-actin. N = 5/group. Data in the graph are shown as mean ± SEM. (G) Western blot analysis of CPT1a and PGC1α in the lysates of isolated endothelial cells from the non-diabetic and diabetic kidneys of control and eKO mice. Representative blots are shown here. Densitometry calculations were normalized to β-actin. N = 5 non-diabetic group, N = 6 diabetic group. Data in the graph are shown as mean ± SEM. One-way ANOVA Tukey post hoc test was used for the analysis of statistical significance. ∗p < 0.05.

**Figure 6:**
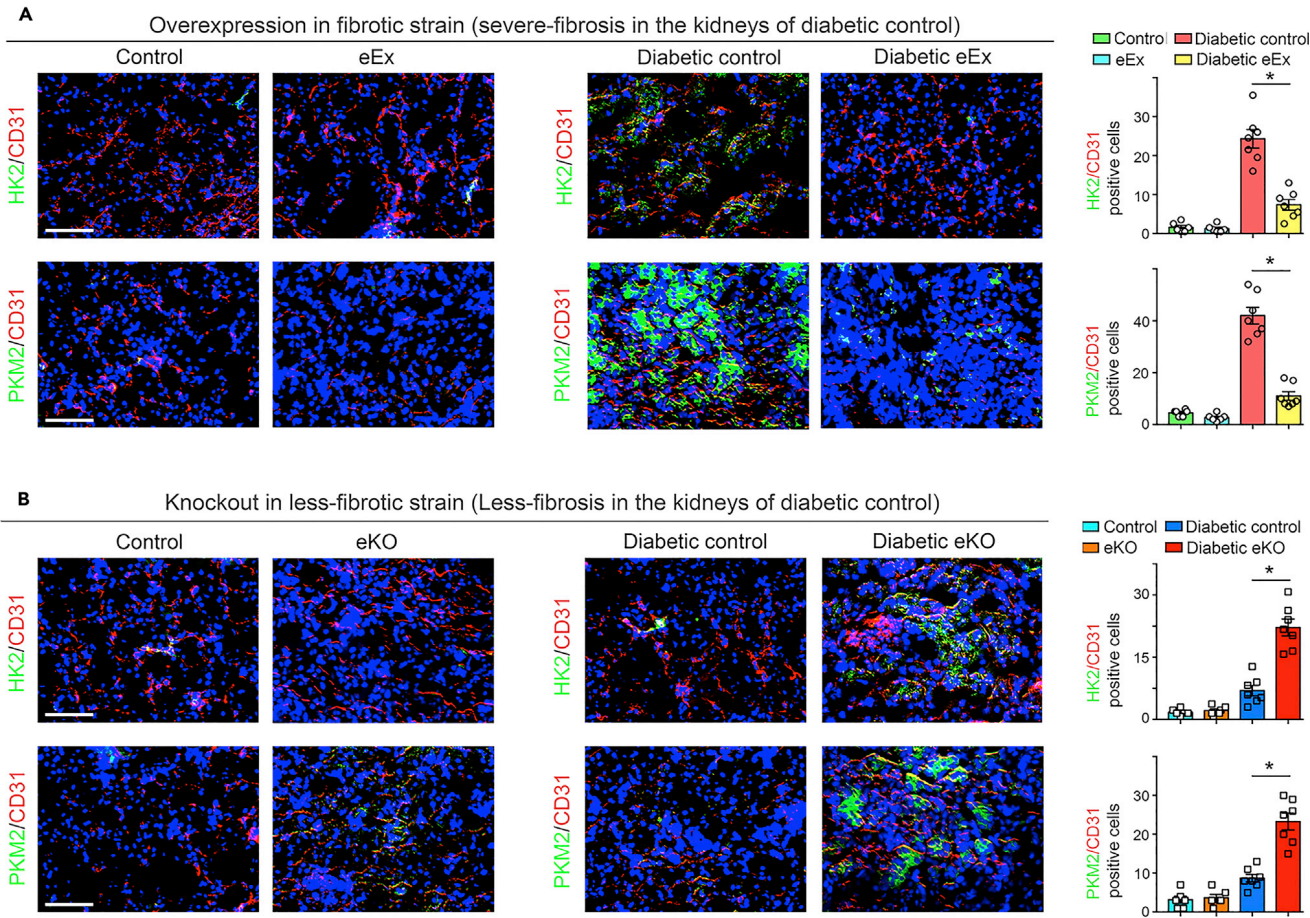
SIRT3 regulates defective glucose metabolism in the kidney endothelial cells (A) Immunofluorescence analysis was performed in the kidneys of non-diabetic and diabetic control and eEx mice by fluorescence microscopy. HK2 and PKM2 protein expression was analyzed in the CD31-positive cells. Merged and representative pictures are shown. N = 5 non-diabetic group, N = 7 diabetic control and diabetic eEx group. (B) Immunofluorescence analysis was performed in the kidneys of non-diabetic and diabetic control and eKO mice by fluorescence microscopy. HK2 and PKM2 protein expression was analyzed in the CD31-positive cells. Merged and representative pictures are shown. N = 5 non-diabetic group, N = 7 for diabetic control and diabetic eKO group. Scale bar, 50 mm in each panel. 40× images are shown. Data in the graph are shown as mean ± SEM. One-way ANOVA Tukey post hoc test was used for the analysis of statistical significance. ∗p < 0.05.

We isolated ECs from the kidneys of diabetic and non-diabetic mice (Figure 5A). The ECs isolated from diabetic eEx exhibited suppressed levels of αSMA, TGFβR1, smad3 phosphorylation, hexokinase 2 (HK2), pyruvate kinase M2 type (PKM2), PKM2 dimer interconversion, and pyruvate dehydrogenase kinase 4 (PDK4) when compared with diabetic controls; however, ECs from kidneys of diabetic eKO displayed higher levels of αSMA, TGFβR1, smad3 phosphorylation, HK2, PKM2 and PKM2 dimer interconversion, and PDK4 when compared with diabetic littermate controls (Figures 5B–5E). The isolated cells from diabetic eEx exhibited suppressed levels of HIF1α and higher level of HIF1α hydroxylation when compared with diabetic control littermates; however, isolated cells from kidneys of diabetic eKO displayed higher levels of HIF1α and reduced level of HIF1α hydroxylation when compared with diabetic littermate controls (Figures S8A and S8B). The isolated cells from diabetic eEx exhibited suppressed levels of dynamin-related protein 1 (drp1) and higher level of mitofusin 2 (mfn2) when compared with diabetic control littermates; however, isolated cells from kidneys of diabetic eKO displayed higher levels of drp1 and reduced level of mfn2 when compared with diabetic littermate controls (Figures S8C and S8D). The ECs isolated from diabetic eEx exhibited higher level of CPT1a and PGC1α when compared with diabetic controls; however, ECs from kidneys of diabetic eKO displayed suppressed levels of CPT1a and PGC1α when compared with diabetic controls (Figures 5F and 5G). The isolated cells from diabetic eEx exhibited suppressed hexokinase, phosphofructokinase enzyme activities, and lactate level, whereas an elevated intracellular ATP levels, when compared with diabetic control littermates; however, isolated cells from diabetic eKO displayed higher hexokinase, phosphofructokinase enzyme activities, and lactate level, whereas an reduced intracellular ATP levels, when compared with diabetic control littermates (Figures S9 and S10). While analyzing immunofluorescent staining, we observed lower expression of glycolytic enzymes in CD31-positive cells in the kidney of diabetic eEx (Figure 6A) and higher protein expression levels in CD31-positive cells of diabetic eKO kidney when compared with diabetic littermate controls (Figure 6B).

### SIRT3 deficiency disrupts metabolic homeostasis in cultured endothelial cells

**Figure 7:**
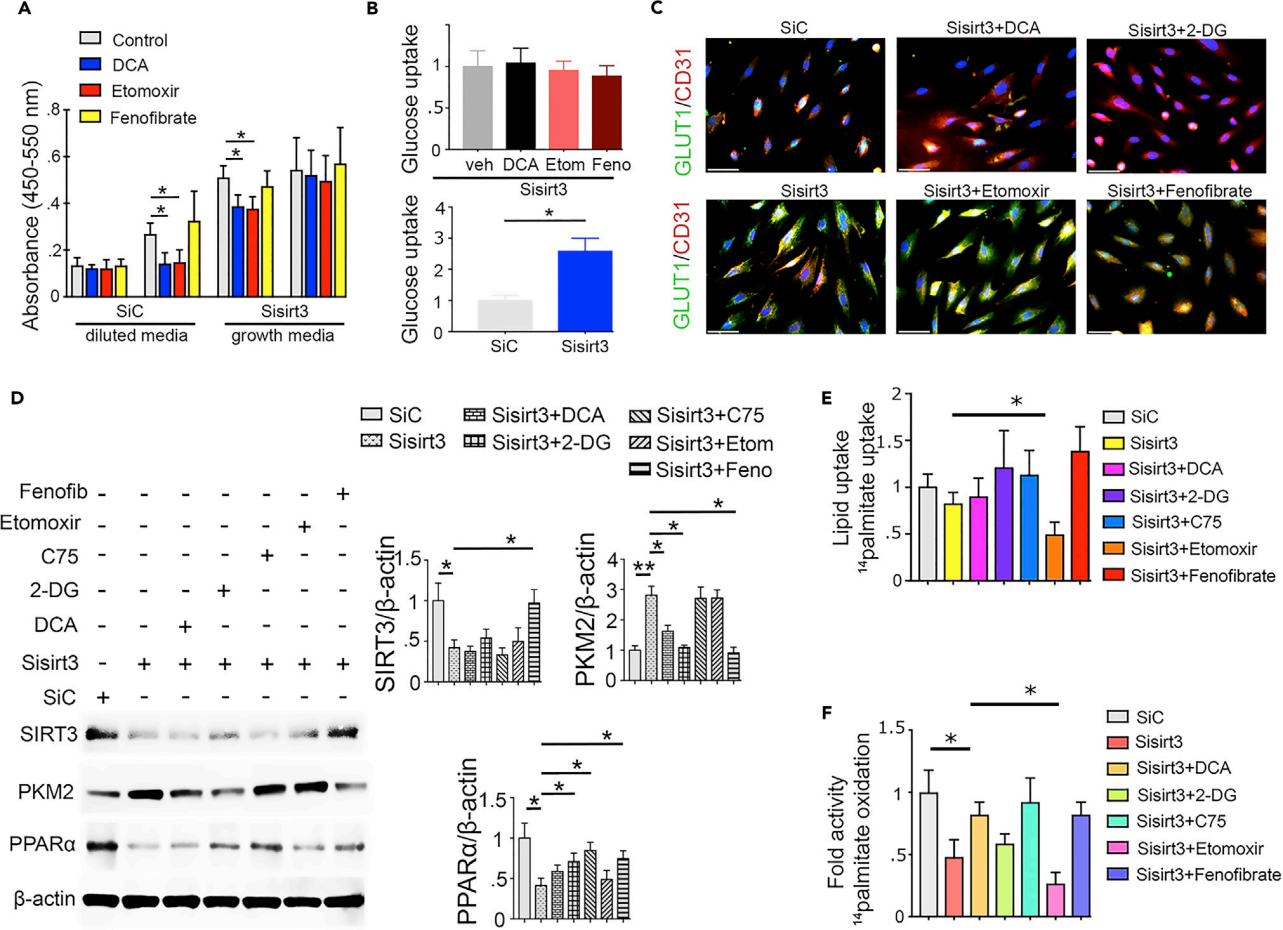
SIRT3 deficiency disrupts metabolic homeostasis in endothelial cells Brdu cell proliferation assay in control siRNA- and Sirt3 siRNA-transfected HUVECs. Treatment with DCA (glycolysis inhibitor), etomoxir (CPT1a inhibitor), and fenofibrate (PPARα agonist) in control siRNA- and Sirt3 siRNA-transfected HUVECs. Three independent sets of experiments were performed. (B) Glucose uptake assay in the experimental groups was analyzed by fluorimetric method. Three independent sets of experiments were performed. (C) GLUT1 translocation from cytoplasm to cell membrane (using CD31 as an endothelial cell marker) in the experimental groups was analyzed using immunofluorescence. GLUT1, green: FITC; CD31, red: rhodamine, and DAPI: blue. Scale bar, 50 μm. (D) Western blot analysis of SIRT3, PKM2, and PPARα in SIRT3 siRNA knockdown cells treated with glycolysis inhibitors (DCA and 2-DG); fatty acid modulators, i.e., etomoxir (CPT1a inhibitor); C75 (fatty acid synthase inhibitor); and fenofibrate (PPARα agonist). Representative blots from four blots are shown. Densitometry analysis by ImageJ. The data in the each graph are normalized by β-actin. (E) Measurement of fatty acid uptake by radioactivity incorporation using ^14^C palmitate in 4 h serum-starved HUVECs. Samples in tetraplicate were analyzed. CPM were counted and normalized to protein. (F) ^14^C palmitate oxidation measured by ^14^CO_2_ release. CPM were counted and normalized to the protein in the well. Samples in tetraplicate were analyzed. Data in the graph are shown as mean ± SEM. One-way ANOVA Tukey post hoc test was used for the analysis of statistical significance. ∗p < 0.05.

To analyze the metabolic alterations specific to SIRT3, we knocked down SIRT3 in ECs. While culturing the SIRT3-depleted cells in growth media, glycolysis inhibitor (dichloroacetate [DCA]) and CPT1a inhibitor (etomoxir) did not alter the level of cell proliferation (Figure 7A); however, culturing the SIRT3-depleted cells in diluted serum media, we observed that DCA and etomoxir caused significant reduction in the level of cell proliferation (Figure 7A). Moreover, we found that SIRT3-deficient cells had a higher level of glucose uptake; glycolysis inhibitors did not alter the level of glucose uptake in SIRT3-deficient cells (Figure 7B). The level of GLUT1 translocation from the cytosol to the cell membrane was also higher in SIRT3-depleted cells (Figure 7C).

To probe the effect of glycolysis inhibition on TGFβ2-induced induction of mesenchymal transformation, we treated DCA with or without TGFβ2-stimulated human umbilical cord endothelial cells (Figure S11A). TGFβ2 stimulation diminished the CD31 and SIRT3, while causing concomitant induction of αSMA and TGFβR1; DCA treatment restored the CD31 and SIRT3 protein levels, with significant suppression of the levels of αSMA and TGFβR1 (Figure S11A).

TGFβ2 did not alter the level of ^14^C palmitate uptake (Figure S11B). TGFβ2 reduced the level of ^14^C palmitate oxidation; glycolysis inhibitors and fatty acid oxidation (FAO) activator (C75) restored the level of FAO in the TGFβ2-treated cells (Figure S11C).

Moreover, western blot analysis of key metabolic regulators in SIRT3-deficient cells revealed significant suppression of PPARα and induction of PKM2. Glycolysis inhibition did not restore SIRT3 protein level; however, it suppressed the PKM2 levels and elevated PPARα in the SIRT3-depleted cells. Fatty acid modulators (C75 and etomoxir) did not alter SIRT3 and PKM2 level, whereas fenofibrate significantly restored the level of SIRT3 and reduced the PKM2 level in the SIRT3-depleted cells (Figure 7D). C75 restored PPARα level in the SIRT3-depleted cells (Figure 7D).

Moreover, SIRT3-depleted cells did not alter the level of lipid uptake, whereas it displayed significant suppression in the level of FAO (Figures 7E and 7F). Glycolysis inhibition did not restore the level of FAO, whereas etomoxir suppressed the level of FAO in SIRT3-deficient cells. C75 restored the suppression in FAO (Figures 7E and 7F), suggesting that SIRT3-deficient ECs reprogram the central metabolism for their survival.

### SIRT3 deficiency-linked EndMT induces mesenchymal transformation in renal tubular epithelial cells

**Figure 8:**
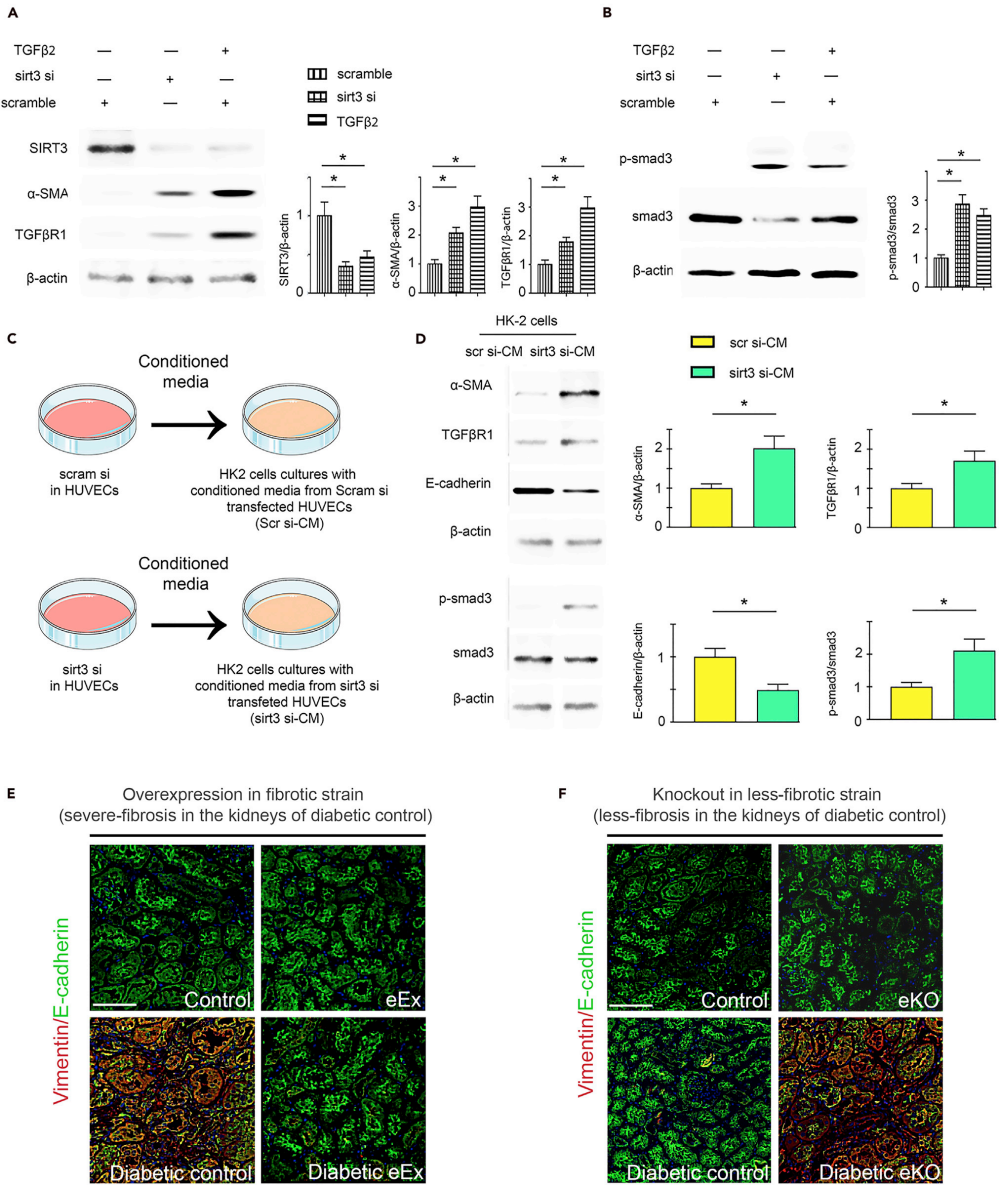
SIRT3 deficiency in endothelial cells causes mesenchymal activation in renal tubular epithelial cells (A) Western blot analysis of SIRT3, α-SMA, and TGFβR1 in the scramble siRNA, sirt3 siRNA-transfected, and TGFβ2-stimulated HUVECs. (B) Smad3 phosphorylation and total smad3 in the scramble siRNA, sirt3 siRNA-transfected, and TGFβ2-stimulated HUVECs. Representative blots are shown. Densitometry calculations are normalized by β-actin. Three independent experiments were performed. (C) Design of conditioned media experiment. Renal tubular epithelial cells (HK2 cells) were cultured in the conditioned media either from scramble siRNA- or sirt3 siRNA-transfected endothelial cells (HUVECs). (D) Representative western blot images of the indicated molecules from three independent experiments are shown. Densitometric analysis of the levels relative to β-actin is shown. Data in the graph are shown as mean ± SEM. (E) Immunofluorescence analysis for vimentin/E-cadherin in the kidneys of non-diabetic and diabetic control littermates and eEx mice. Representative pictures are shown. N = 7 per group. Scale bar, 50 μm. (F) Immunofluorescence analysis for vimentin/E-cadherin in the kidneys of non-diabetic and diabetic control littermates and eKO mice. Representative pictures are shown. N = 7 per group. Scale bar, 50 μm. Data in the graph are shown as mean ± SEM. One-way ANOVA Tukey post hoc test was used for the analysis of statistical significance. ∗p < 0.05.

Western blot analysis revealed that reprogrammed SIRT3-depleted cells had induced the level of αSMA, TGFβR1, and smad3 phosphorylation (Figures 8A and 8B). To test the contribution of reprogrammed SIRT3-depleted cells on the mesenchymal activation in tubular epithelial cells, we transferred the conditioned media either from SIRT3-replete or SIRT3-depleted HUVECs to renal tubular epithelial cells (HK2 cells) (Figure 8C). The SIRT3-depleted cells-conditioned media (sirt3 si-CM) treatment caused significant suppression of E-cadherin levels; however, it caused induction of αSMA, TGFβR1, and smad3 phosphorylation protein when compared with SIRT3 replete cells-conditioned media (scramble si-CM)-treated HK2 cells (Figure 8D). The SIRT3-depleted cells-conditioned media (sirt3 si-CM) treatment caused significant elevation in the proinflammatory IL-1β levels when compared with SIRT3 replete cells-conditioned media (scramble si-CM)-treated HK2 cells (Figure S12). To investigate whether endothelial SIRT3 influences EMT in the kidney, we examined E-cadherin/vimentin (EMT events) co-labeling in the diabetic kidneys. The kidneys of diabetic eEX exhibited suppressed levels of vimentin in E-cadherin-positive cells when compared with diabetic controls, whereas the kidneys of diabetic eKO displayed significantly higher levels of vimentin in E-cadherin-positive cells when compared with diabetic littermate controls (Figures 8E and 8F).

## Discussion

We here describes the crucial role of endothelial SIRT3 in the regulation of fibrogenic processes in the mouse model of DKD. We describe (1) the loss of endothelial SIRT3 and overexpressed level of endothelial SIRT3 in the renal vasculature, functions, and fibrogenic processes and (2) regulation of endothelial SIRT3 on central metabolic processes, which affect activation of fibrogenic processes in the diabetic kidneys. Our results demonstrate that endothelial SIRT3 regulates glucose and lipid metabolism, and associated mesenchymal trans-differentiation process, by maintaining control over TGFβ-Smad3 signaling in the kidneys of diabetic mice. SIRT3 deficiency is one of the fibrotic phenotypes in diabetes that leads to PKC activation and PKM2 tetramer-to-dimer interconversion; ultimately, these processes alter the metabolic switch toward defective metabolism and associated mesenchymal activation in renal epithelial cells (Li et al., 2020b; Srivastava et al., 2018, 2020c).

In addition, it is evident from our results that endothelial SIRT3 is a critical antifibroic molecule in diabetic kidneys. Our data demonstrate that SIRT3 suppression in the kidney ECs is the critical step for the metabolic reprogramming and contributes to fibrogenic events. To test the contribution of SIRT3 in EC homeostasis, we bred the endothelial SIRT3 overexpression mouse (knockin) and endothelial SIRT3 knockout mouse. It is clear from the results that overexpression of SIRT3 in ECs attenuates fibrosis by mitigating the disrupted metabolism-linked EndMT in the kidney of diabetic mice. Our results further strengthen our views when we analyzed the loss of function of SIRT3 protein, which clearly demonstrates that loss of SIRT3 in ECs worsens fibrogenic processes, displays higher level of TGFβ-smad3 signaling, and displays defective metabolism-associated-EndMT, suggesting that loss of SIRT3 disrupts the EC homeostasis and accelerates the fibrogenic processes in the diabetic kidney. Taken together, these demonstrate that SIRT3 is a critical molecule in EC metabolism and regulates EndMT in the kidney.

To envisage deeper the contribution of defective metabolism in the ECs, we used the small chemicals (which are well known to modulate the glucose and fatty acid metabolism) in the cultured HUVECs. The results from the cultured cells confirm that SIRT3-depleted cells require a high rate of glycolysis and a higher level of FAO for their survival. Of note, PKM2 tetramer-to-dimer interconversion is a well-known regulator of central metabolism in ECs (Cantelmo et al., 2016; De Bock et al., 2013; Kim et al., 2018; Schoors et al., 2014; Zhou et al., 2019). Our data suggest that SIRT3 in the ECs regulates PKM2 tetramer-to-dimer interconversion and linked disruption in central metabolism in diabetic kidney. Moreover, the data clearly demonstrate that SIRT3-depleted cells have higher GLUT1 translocation and higher glucose uptake; consequently, these processes result in the accumulation of glucose inside the cells, which in turns activates the HK2 enzyme in the cytosol. Taken together, our data suggest that cumulative effects of HIF1α and PKM2 dimer lead to defective central metabolism in ECs and that SIRT3 regulates HIF1α through hydroxylation and PKM2 dimer by converting into PKM2 tetramer.

To begin to understand how SIRT3 deficiency-linked EndMT induces mesenchymal transformation in renal epithelial cells, we transferred the conditioned media from SIRT3-depleted ECs to cultured renal tubular HK-2 cells. Interestingly, we observed the gain of mesenchymal markers, activation of TGFβ-smad3 signaling, and concomitant suppression of epithelial cell markers. These findings suggest that EndMT leads to the mesenchymal activation program (EMT) in renal tubular cells. EndMT-mediated EMT activation is critical in the activation of fibrogenic response in the whole kidneys (Li et al., 2020a; Srivastava et al., 2020d).

Current therapeutic regimens for patients who have symptoms of DKD include angiotensin-converting enzyme inhibitors, angiotensin II receptor type 1 blockers (ARBs), and statins that can retard, but not prevent, the progression of incidence of end-stage kidney disease in diabetes. However, side effects and intolerance to these agents often exceed their overall efficacy. The results from our data make it clear that ameliorating the level of SIRT3 in the EC can be a future strategy in combating diabetes-associated kidney fibrosis.

In conclusion, our findings demonstrate the functional importance of SIRT3 protein in EC homeostasis and highlight the regulatory role of SIRT3 on EndMT in diabetic kidneys, mediated by control over TGFβ-smad3 signaling and linked-defective metabolism. SIRT3 disrupts the metabolic reprogramming in endothelial-derived fibroblasts. The SIRT3-deficient EndMT cells activate the mesenchymal transformations in epithelial cells (through EMTs) in a paracrine manner by releasing IL-1β. This study provides new insight into the biology of SIRT3 and its regulation on EC homeostasis in the kidney.

### Limitations of the study

Although we observed severe fibrosis in the diabetic kidneys of SIRT3-deficient mice (belonging to the C57BL/6 background), the study of fibrogenic phenotype in overexpression mice (Tie1+SIRT3 tg, of C57BL/6 background) was limited because the diabetic kidneys of C57BL/6 mice show limited fibrogenesis upon diabetes induction. Therefore, to envisage a clear impact of overexpression, we bred in fibrotic mouse background (CD-1 mouse background) until ninth generation. Till date, there is no suitable model for DKD in the C57BL/6 mouse background that shows severe fibrosis in kidneys. Our data highlight the regulatory role of SIRT3 in the ECs; however, it is still not clear how mitochondrial SIRT3 regulates glycolysis in the cytosol or how cytosolic or nuclear SIRT3 regulates mitochondrial metabolism in the ECs and/or what are impacts of intracellular SIRT3 translocations in the metabolic shift in ECs. It will be interesting to study the role of upstream regulators of SIRT3 in different cellular compartmentations that might have a significant effect on disease phenotype in the kidney. We believe that further studies will be required to understand the metabolic communications in kidney disease pathogenesis.

### Resource availability

#### Lead contact

Further information and requests for resources should be directed to and will be fulfilled by the lead contact, Swayam Prakash Srivastava, Yale University United States (swayam.cdri@gmail.com; swayam.srivastava@yale.edu).

#### Materials availability

This study did not generate new unique materials.

#### Data and code availability

All data produced or analyzed for this study are included in the published article and its supplementary information files.

## Methods

All methods can be found in the accompanying transparent methods supplemental file.

## Supplemental information

Document S1. Transparent methods and Figures S1–S12
