# Supplementary material for: Endothelial SIRT3 regulates myofibroblast metabolic shifts in diabetic kidneys
Source: iScience. 2021 Apr 6;24(5):102390. doi: 10.1016/j.isci.2021.102390 (PMC8086030; doi:10.1016/j.isci.2021.102390)
Supplement: Document S1. Transparent methods and Figures S1–S12 [file mmc1.pdf]

iScience, Volume *24*

Supplemental information

Endothelial SIRT3 regulates myofibroblast metabolic shifts in diabetic kidneys

Swayam Prakash Srivastava, Jinpeng Li, Yuta Takagaki, Munehiro Kitada, Julie E. Goodwin, Keizo Kanasaki, and Daisuke Koya

Supplemental Information

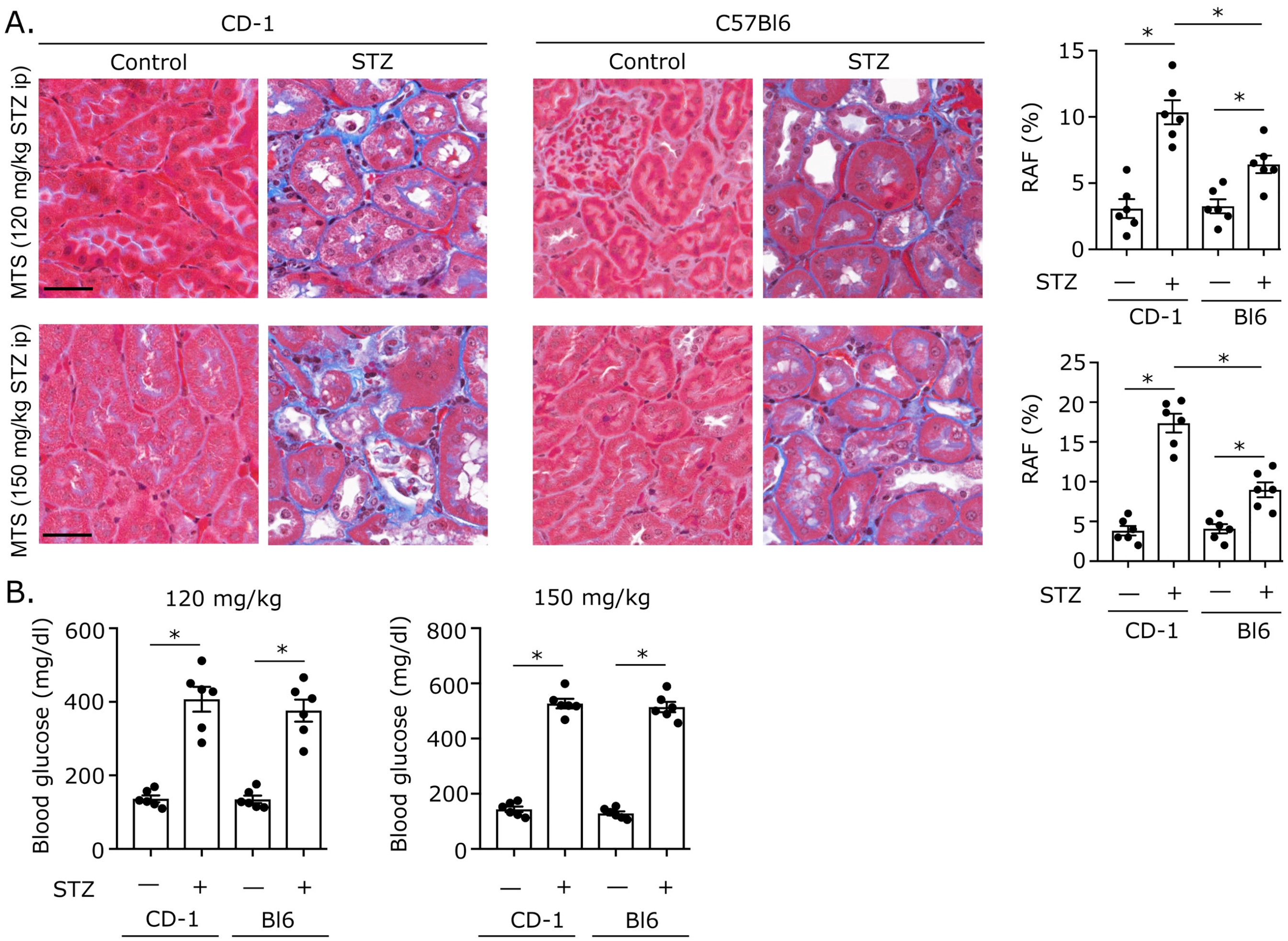

Figure S1. Dose dependent effect of streptozotocin on the renal fibrogenic phenotype of CD-1 and C57Bl6 mice, Related to Figure 1. **(a)** Masson trichrome staining in the kidneys of non-diabetic and diabetic CD-1 and C57Bl6 mice. Representative images are shown here. Area of fibrosis (%) was measured using the ImageJ program. N=6/each group. Data in the graph are shown as mean ± SEM. Scale bar: 50 µm. **(b)** Blood glucose. First panel at (120 mg/kg STZ i.p. dose) and second panel at (150 mg/kg STZ i.p. dose). N=6/each group. Data in the graph are shown as mean ± SEM. One-way ANOVA Tukey post hoc test was used for the analysis of statistical significance. Significance *-<0.05.

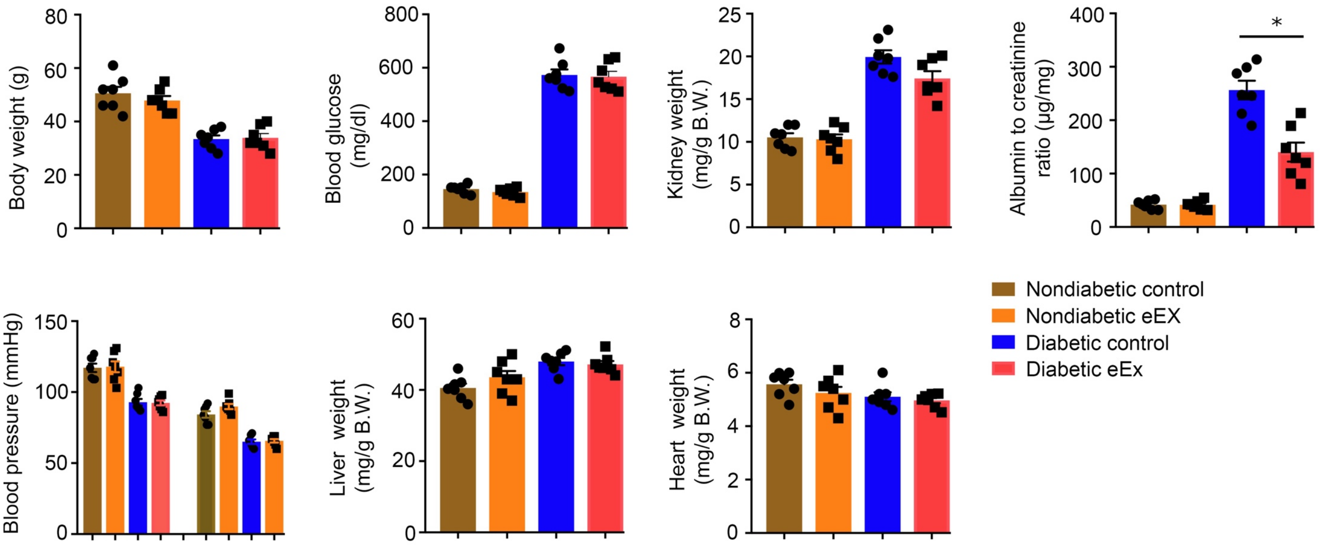

Figure S2 Physiological characteristics of non-diabetic and diabetic, eEx mice and littermates control, Related to Figure 2. Body weight, blood glucose, kidney weight, albumin-to-creatinine ratio (ACR), blood pressure, liver weight and heart weight were measured. N=7/each group. Data in the graph are shown as mean ± SEM. One-way ANOVA Tukey post hoc test was used for the analysis of statistical significance. Significance *-<0.05.

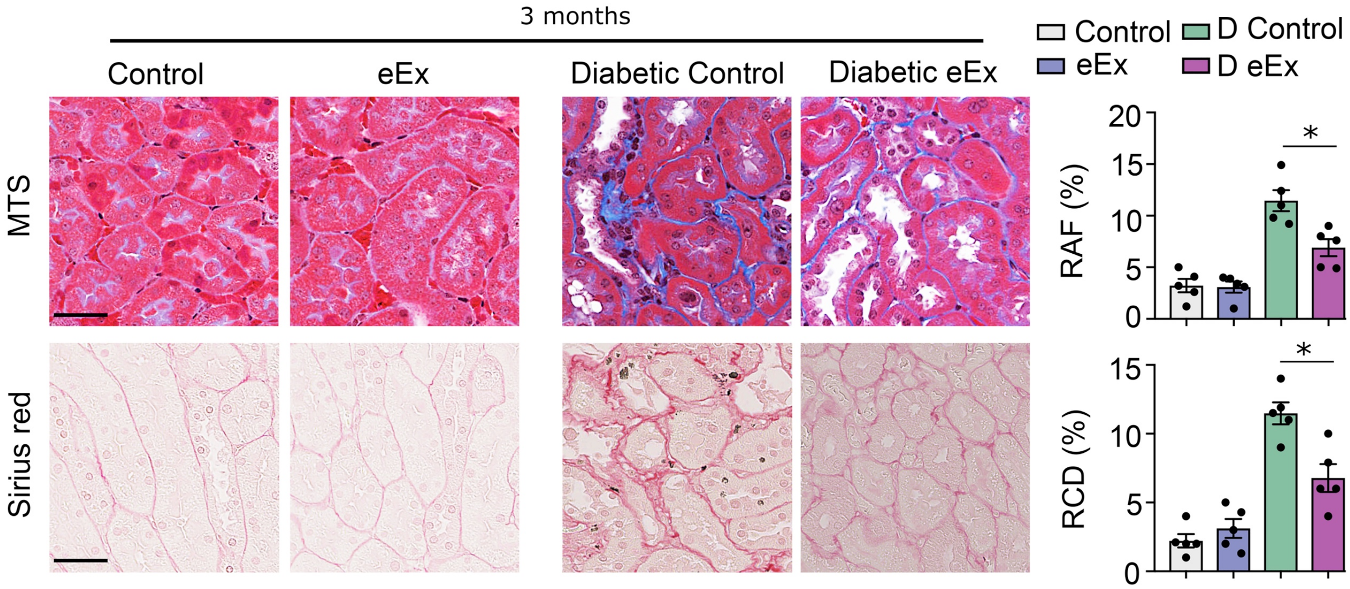

Figure S3 Renal fibrogenic analysis of endothelial SIRT3 overexpressed (eEx) mice and control littermates after 3 months of diabetes induction, Related to Figure 2. Masson trichrome and Sirius red staining in the kidneys of non-diabetic and diabetic eEx and control mice. Representative images are shown here. MTS-relative area of fibrosis (RAF in %) and Sirius red-relative collagen deposition (RCD in %) were measured using the ImageJ program. N=5/each group. Scale bar: 50 µm. Data in the graph are shown as mean ± SEM. One-way ANOVA Tukey post hoc test was used for the analysis of statistical significance. Significance *-<0.05.

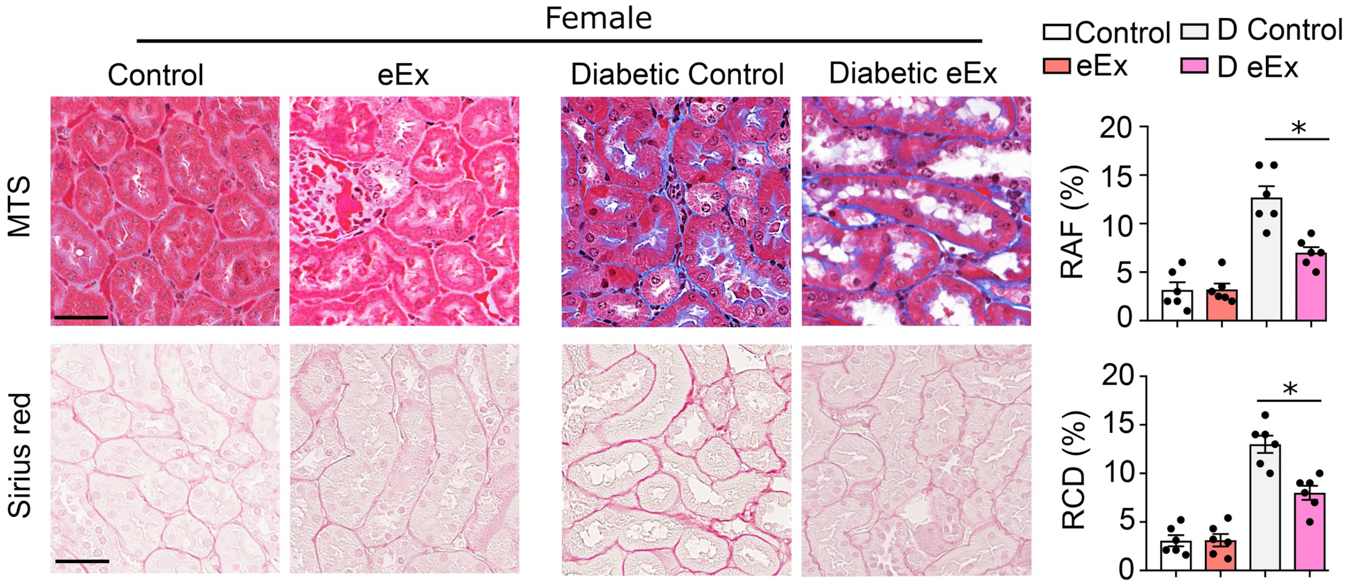

Figure S4 Renal fibrogenic analysis in female endothelial SIRT3 overexpressed (eEx) mice and control littermates, Related to Figure 2. Masson trichrome and Sirius red staining in the kidneys of non-diabetic and diabetic female eEx and female control mice. Representative images are shown here. MTS-relative area of fibrosis (RAF in %) and Sirius red-relative collagen deposition (RCD in %) were measured using the ImageJ program. N=6/each group. Scale bar: 50 μm. Data in the graph are shown as mean ± SEM. One-way ANOVA Tukey post hoc test was used for the analysis of statistical significance. Significance *-<0.05.

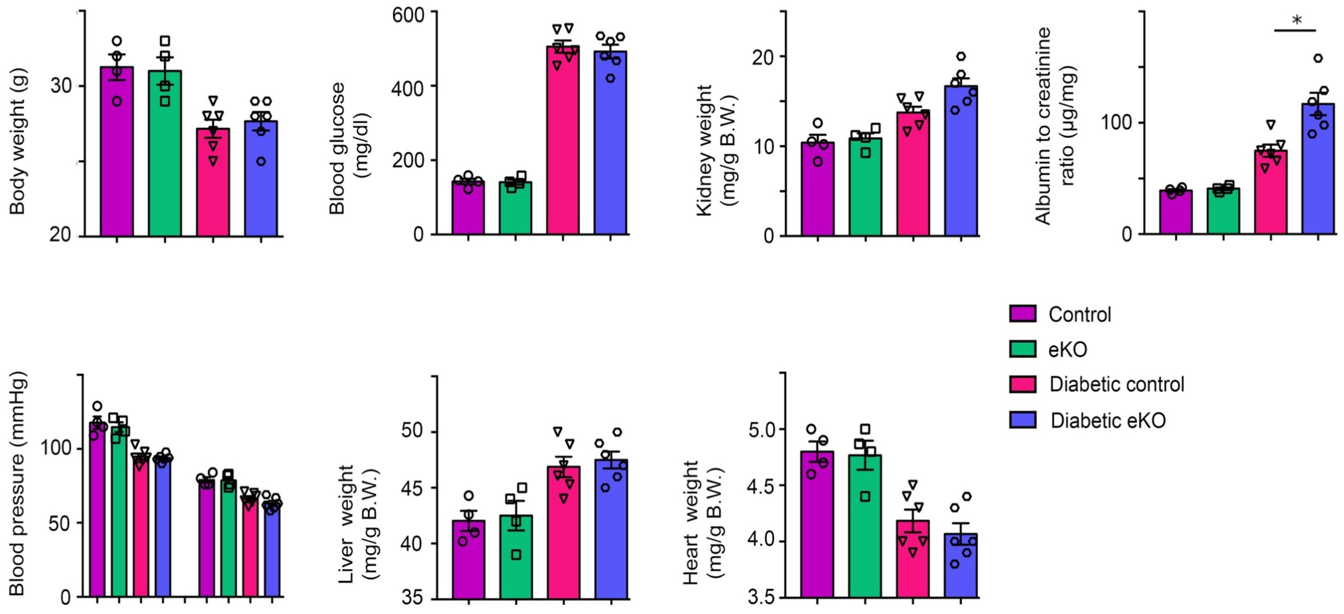

Figure S5 Physiological characteristics of non-diabetic and diabetic, eKO mice and control littermates, Related to Figure 3. Body weight, blood glucose, kidney weight, ACR, blood pressure, liver weight and heart weight were measured. N=4 for non-diabetic, N=6 for diabetic control and for diabetic eEx mice. Data in the graph are shown as mean ± SEM. One-way ANOVA Tukey post hoc test was used for the analysis of statistical significance. Significance *-<0.05.

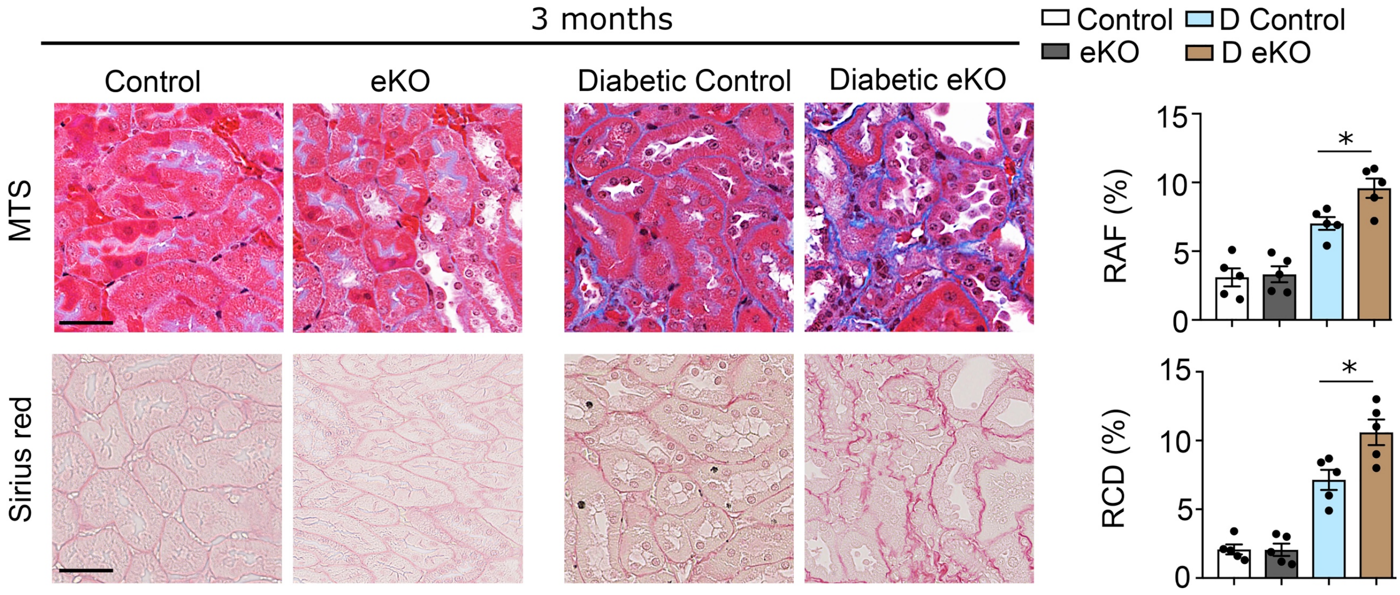

Figure S6 Renal fibrogenic analysis of endothelial SIRT3 knock out (eKO) mice and control littermates after 3 months of diabetes induction, Related to Figure 3. Masson trichrome and Sirius red staining in the kidneys of non-diabetic and diabetic eKO and control mice. Representative images are shown here. MTS-relative area of fibrosis (RAF in %) and Sirius red-relative collagen deposition (RCD in %) were measured using the ImageJ program. N=5/each group. Scale bar: 50 µm. Data in the graph are shown as mean ± SEM. One-way ANOVA Tukey post hoc test was used for the analysis of statistical significance. Significance *-<0.05.

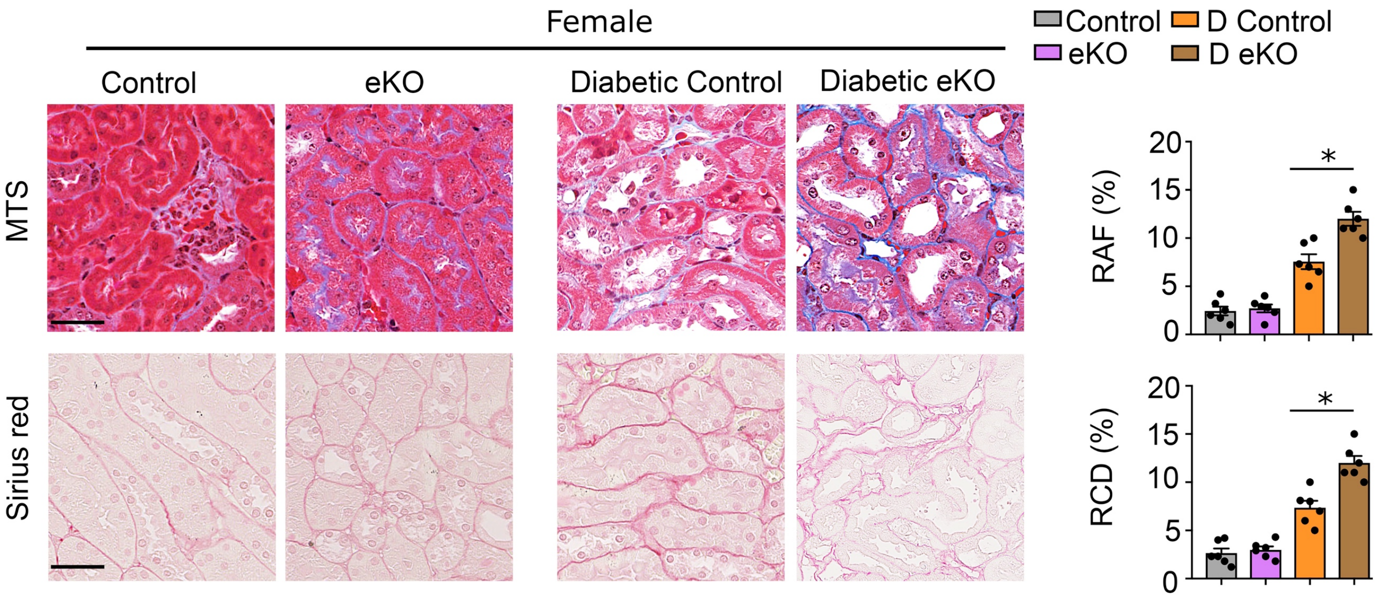

Figure S7 Renal fibrogenic analysis in female endothelial SIRT3 knockout (eKO) mice and control littermates, Related to Figure 3. Masson trichrome and Sirius red staining in the kidneys of non-diabetic and diabetic female eKO and female control mice. Representative images are shown here. MTS-relative area of fibrosis (RAF in %) and Sirius red-relative collagen deposition (RCD in %) were measured using the ImageJ program. N=6/each group. Scale bar: 50 μm. Data in the graph are shown as mean ± SEM. One-way ANOVA Tukey post hoc test was used for the analysis of statistical significance. Significance *-<0.05.

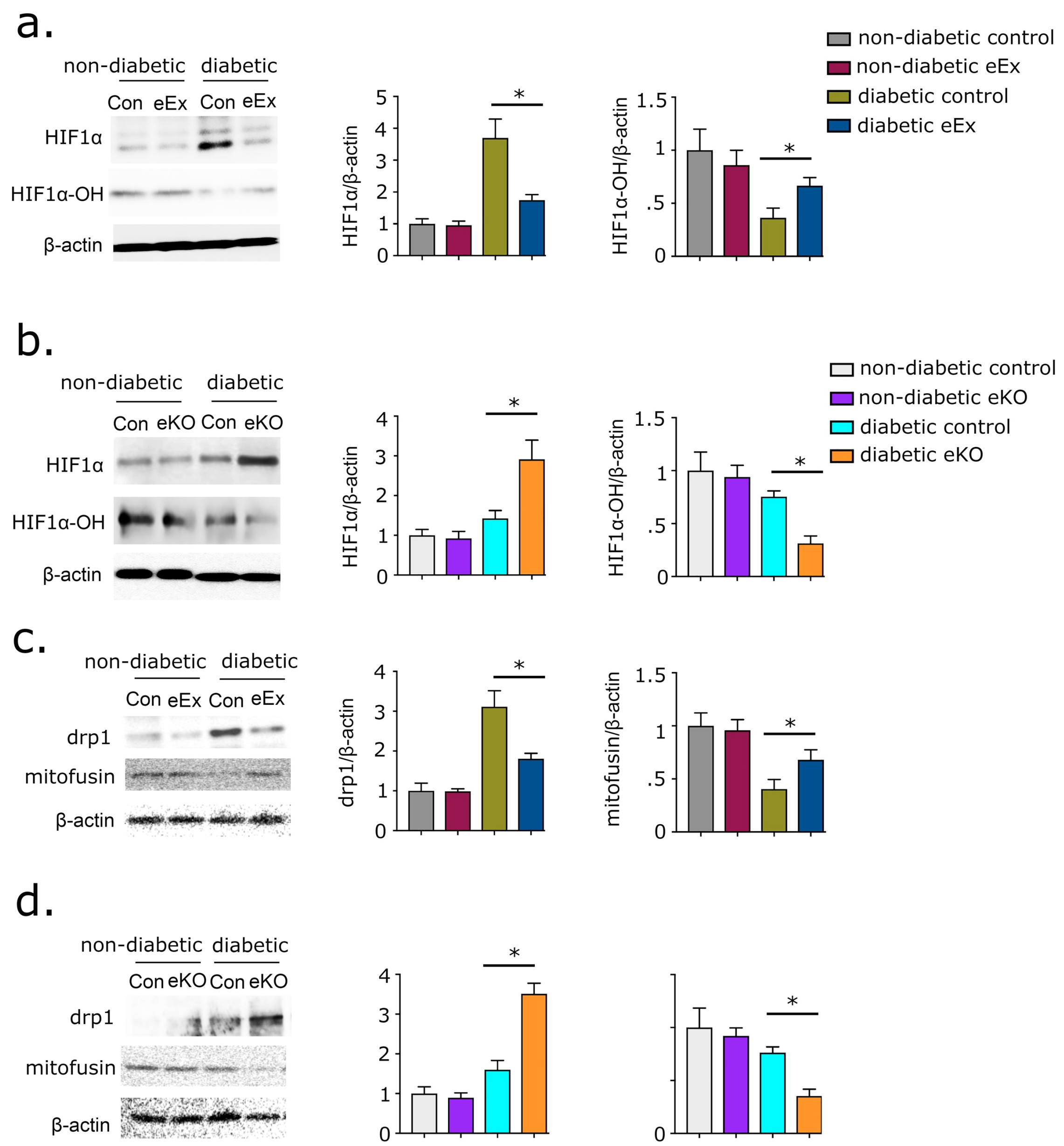

Figure S8 SIRT3 regulates HIF1α hydroxylation in the endothelial cells-derived fibroblasts in kidney, Related to Figure 5. **(a)** Western blot analysis of HIF1α, and HIF1-OH in the lysates of isolated endothelial cells from non-diabetic and diabetic kidneys of control and eEx mice. Representative blots are shown. Densitometry calculations were normalized to β-actin. N=6 were analyzed in each group. **(b)**- Western blot analysis of HIF1α, and HIF1-OH in the lysates of isolated endothelial cells from non-diabetic and diabetic kidneys of control and eKO mice. Representative blots are shown. Densitometry calculations were normalized to β-actin. N=6 were analyzed in each group. **(c)** Western blot analysis of drp1, and mfn2 in the lysates of isolated endothelial cells from non-diabetic and diabetic kidneys of control and eEx mice. Representative blots are shown. Densitometry calculations were normalized to β-actin. N=6 were analyzed in each group. **(d)**- Western blot analysis of drp1, and mfn2 in the lysates of isolated endothelial cells from non-diabetic and diabetic kidneys of control and eKO mice. Representative blots are shown. Densitometry calculations were normalized to β-actin. N=6 were analyzed in each group. Data in the graph are shown as mean ± SEM. One-way ANOVA Tukey post hoc test was used for the analysis of statistical significance. Significance *-<0.05.

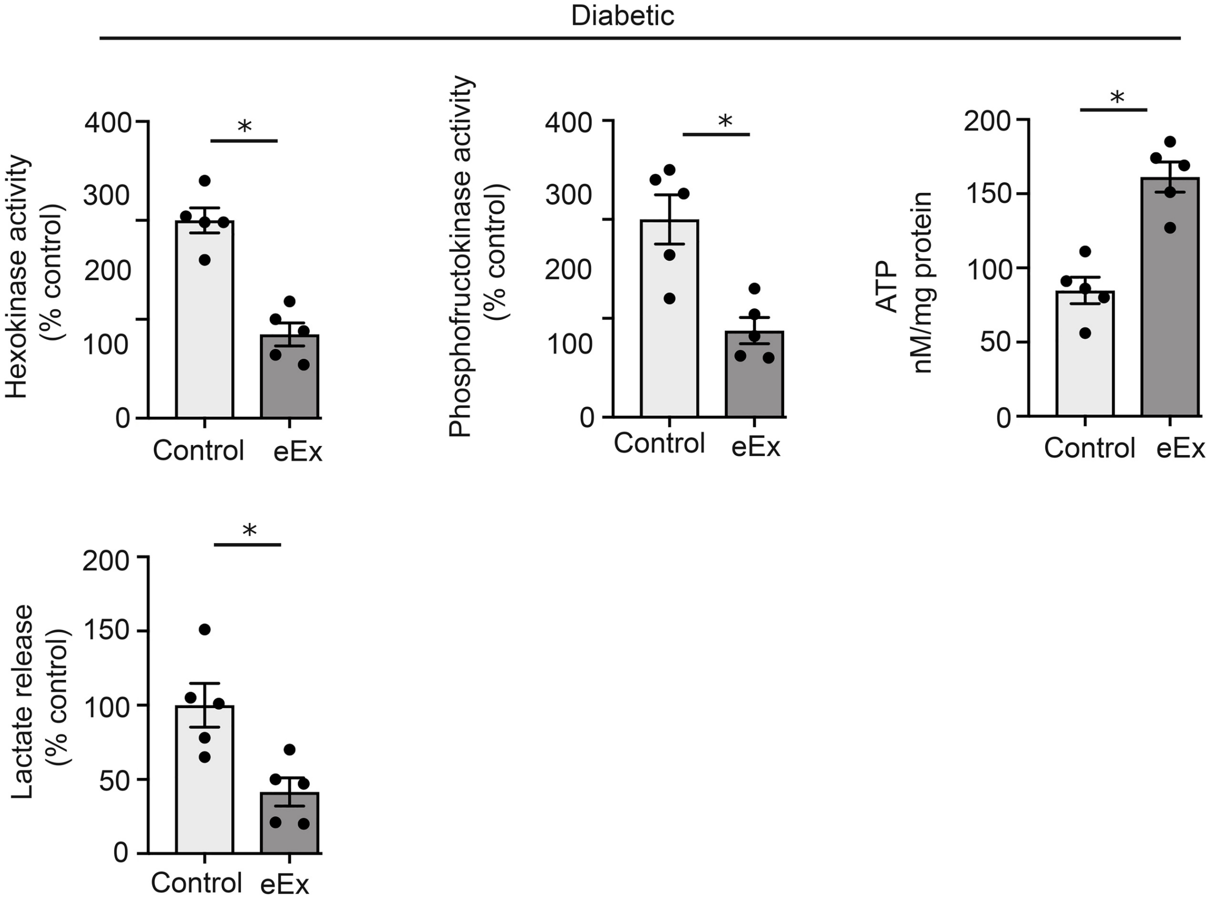

Figure S9 Overexpression of SIRT3 suppressed defective central metabolism in isolated endothelial cells from diabetic mice, Related to Figure 5.

Hexokinase, Phosphofructokinase enzyme activities, ATP level, and lactate release in the media and PPARα transcriptional activity in the isolated endothelial cells from the diabetic control and diabetic eEx. Assays were performed using commercial kits following manufacturer's instructions. N=6/each group were analyzed. Data in the graph are shown as mean ± SEM. Student t-test was used for the analysis of statistical significance. Significance *-<0.05.

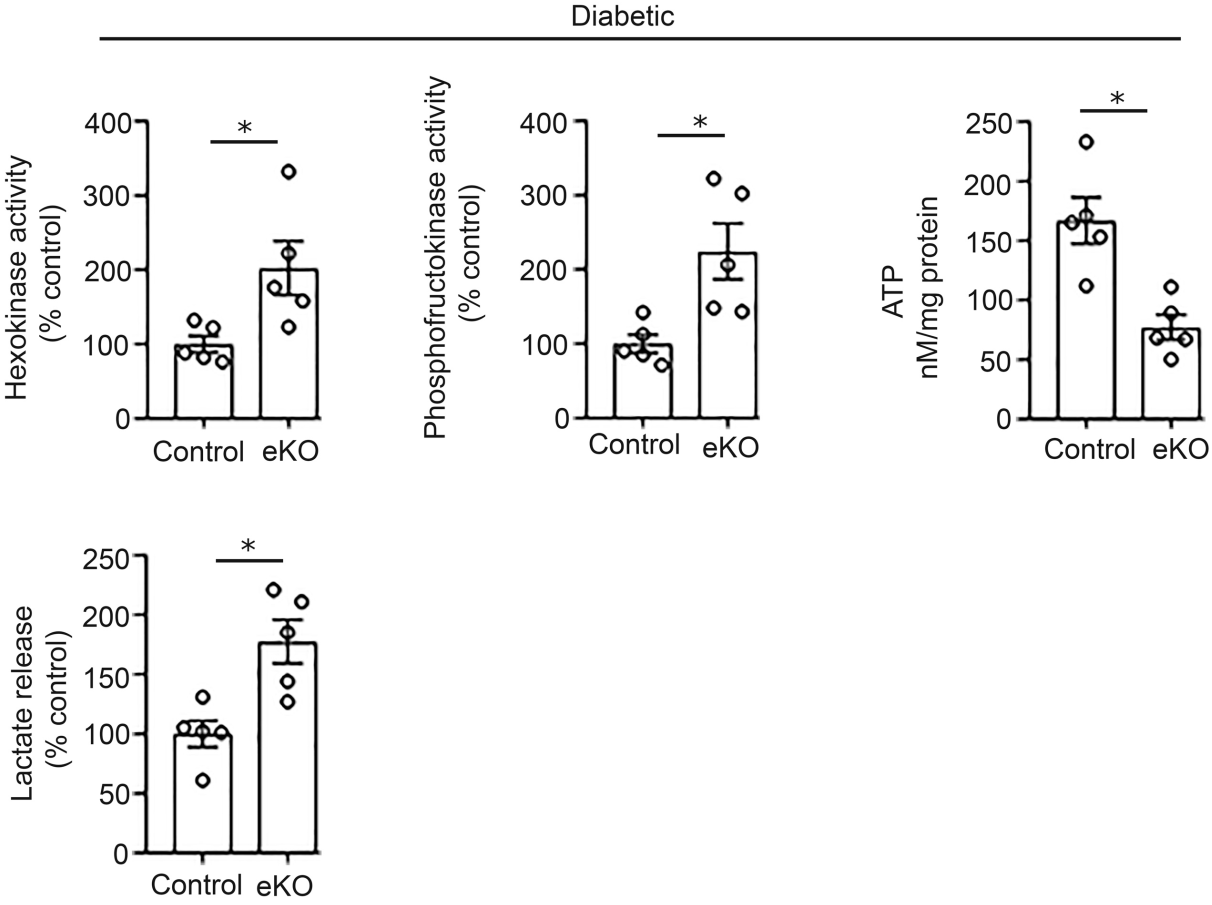

Figure S10 Loss of SIRT3 leads to defective central metabolism in isolated endothelial cells from diabetic mice, Related to Figure 5. Hexokinase, Phosphofructokinase enzyme activities, ATP level, and lactate release in the media and PPARα transcriptional activity in the isolated endothelial cells from the diabetic control and diabetic eKO. Assays were performed using commercial kits following manufacturer's instructions. N=5/each group were analyzed. Data in the graph are shown as mean ± SEM. Student t-test was used for the analysis of statistical significance. Significance *-<0.05.

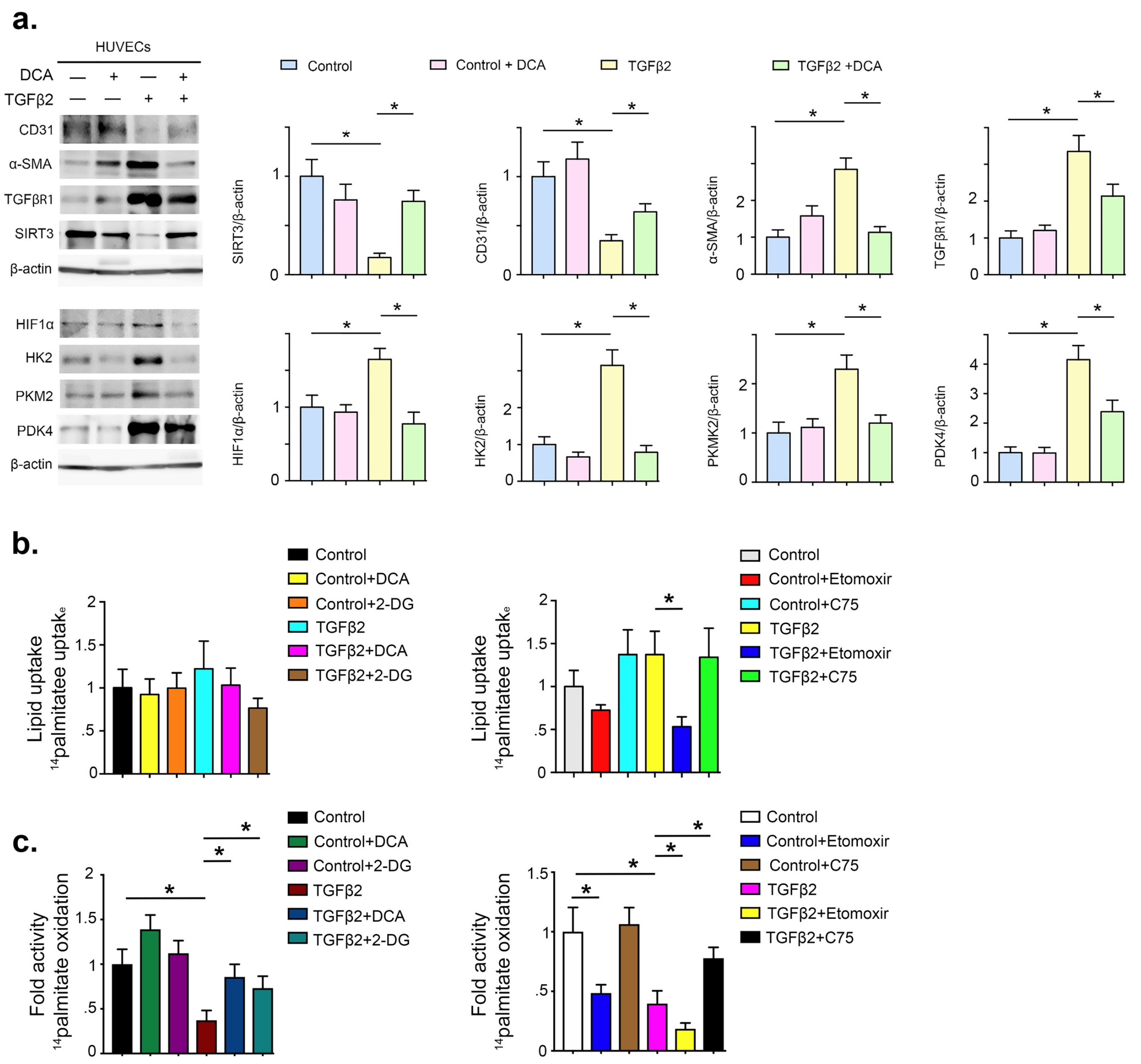

Figure S11 TGFβ2 causes disruption of central metabolism in endothelial cells. Related to Figure 7. **(a)** Western blot analysis of the indicated molecules in HUVEC from five independent experiments is shown. Densitometric analysis of the levels relative to β-actin is shown. Data in the graph are shown as mean ± SEM. **(b)** Measurement of fatty acid uptake by radioactivity incorporation using [^{14}C]-palmitate in glycolysis inhibitors and fatty acid oxidation modulators treated with or without TGFβ2-stimulated HUVECs. Samples in tetraplicate were analyzed. CPM were counted and normalized with protein. (**c**) ^{14}C palmitate oxidation by measuring $^{14}CO_2$ released. CPM were counted and normalized with protein in the well. Samples in tetraplicate were analyzed. Data in the graph are shown as mean ± SEM. Student t-test was used for the analysis of statistical significance. Significance *-<0.05.

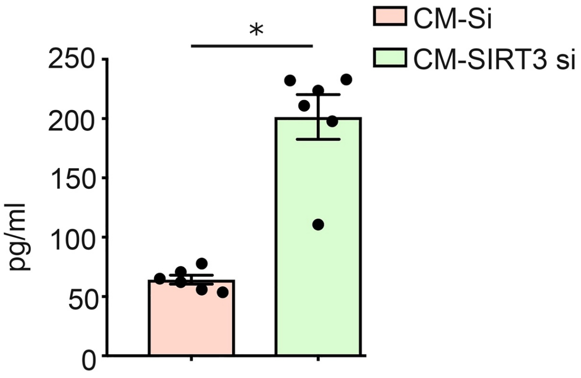

Figure S12 IL-1β level analysis, Related to Figure 8. Determination of IL-1β level in the indicated group. N=6/each group. Data in the graph are shown as mean ± SEM. One-way ANOVA Tukey post hoc test was used for the analysis of statistical significance. Significance *-<0.05.

TRANSPARENT METHODS

Reagent and antibodies

For rabbit polyclonal anti-pyruvate kinase (PK) isozyme M2 (4053s, RRID: AB_1904096), rabbit anti-HK-2 (2867, RRID: AB_2232946), CPT1a (D3B3, 12251) and PGC1α (3G6, 2178) antibodies were purchased from Cell Signaling Technology (Danvers, MA). A mouse monoclonal PDK4 (ab71240) were purchased from Abcam (Cambridge, UK). The goat anti-Sirt3 antibody (sc-365175, RRID: AB_10710522) was purchased from Santa Cruz Biotechnology (Dallas, TX). The mouse monoclonal anti-β-actin (AC-74) (A2228, RRID: AB_476697) antibody was obtained from Sigma (St. Louis, MO, USA). Rabbit polyclonal anti-phospho smad3 (s423 and s425) antibody was purchased from Rockland Immunochemicals (Gilbertsville, PA). Rabbit polyclonal anti-αSMA antibody (GTX100034) was purchased from GeneTex (Irvine, CA). Rabbit polyclonal anti-TGFβR1 antibody (SAB4502958) was obtained from Sigma (St Louis, MO). Fluorescence-, Alexa Fluor 647-, and rhodamine conjugated secondary antibodies were obtained from Jackson ImmunoResearch (West Grove, PA).

Animal Experimentation

The experiments in the methods sections are carried out in accordance with Kanazawa Medical University animal protocols (protocol number 2014-89; 2013-114 and 2014-101), approved by Institutional Animal Care and Use Committee (IACUC). For the gain-of-function studies (SIRT3 over-expression), we utilized transgenic mice with over-expressed levels of SIRT3 in endothelial cells (Tie1-SIRT3 tg mice). We generated Tie1-SIRT3 tg mice in our laboratory and these mice were on the less fibrotic C57BL6 background. These mice were backcrossed for nine generations to transfer the SIRT3 tg gene into the more fibrotic CD-1 mice. We bred Tie1-SIRT3 tg+; CD-1 (eEx) and Tie1-SIRT3 tg-; CD-1 (littermate control). For the loss-of-function of SIRT3 gene, we bred the $SIRT3^{flox/flox}$ mice with VE-Cadherin-Cre mice (eKO) to generate mice with a deletion of the SIRT3 in their endothelial cells. $SIRT3^{flox/flox}$ mice and VE-Cadherin-Cre were purchased from The Jackson laboratory. The VE-Cadherin promoter used to generate the Cre-expressing mice was designed to be expressed predominantly in the endothelial cells.
The induction of diabetes in the CD-1 background mice and C57Bl6 KsJ background mice was performed according to the previously established experimental protocol (Kanasaki et al., 2014; Li et al., 2020; Li et al., 2017; Nagai et al., 2014; Nitta et al., 2016; Shi et al., 2015). In brief, diabetes was induced in 10-week-old male and female eKO mice through 5 multiple consecutive doses of streptozotocin (STZ) at 50 mg/kg i.p. in 10 mmol/L citrate buffer (pH 4.5). However, a single i.p. dose of 200 mg.kg STZ was used to induce diabetes in the male and female eEx mice (eEx mice was in CD-1 mouse background, we followed previously published protocol for induction of diabetes in CD-1 mice (Kanasaki et al., 2014; Shi et al., 2015; Srivastava et al., 2020a, b; Srivastava et al., 2016; Sugimoto et al., 2007). Urine albumin levels were estimated using a Mouse Albumin ELISA Kit (Exocell, Philadelphia, PA).

Morphological Evaluation

We utilized a point-counting method to evaluate the relative area of the mesangial matrix. We analyzed PAS-stained glomeruli from each mouse using a digital microscope screen grid containing 540 (27× 20) points. Masson's trichrome-stained images were evaluated by ImageJ software, and the fibrotic areas were estimated.

Sirius red staining

Deparaffinized sections were incubated with picrosirius red solution for 1 hour at room temperature. The slides were washed twice with acetic acid solution for 30 seconds per wash. The slides were then dehydrated in absolute alcohol three times, cleared in xylene, and

mounted with a synthetic resin. Sirius red staining was analyzed using ImageJ software, and fibrotic areas were quantified.

EndMT detection in vivo
Frozen sections (5 μm) were used for the detection of in vivo EndMT. Cells undergoing EndMT were detected by double-positive labeling for CD31 and αSMA or FSP-1 and TGFβR1. The immune-labeled sections were analyzed by fluorescence microscopy.

Isolation of endothelial cells
Endothelial cells from the kidneys of non-diabetic and diabetic mice were isolated using the kits (Miltenyl Biotech, USA) by following the instructions from manufacturer. In general, kidneys were chopped off into small pieces and prepared for single cells suspension to carry out series of enzymatic reaction by treating with trypsin and Collagenase type I solution. The pellet is dissolved with CD31 magnetic bead and the CD31-labelled cells were separated on the magnetic separator and the cells were further purified on the column provided by manufacturer. Cell number were counted by hemocytometer and were plated on 0.1 gelatin coated Petri dishes.

RNA isolation and qPCR
Total RNA was isolated from the isolated endothelial cells using Qiagen RNeasy Mini Kit (Qiagen, Hilden, Germany). Complementary DNA (cDNA) was generated by using the Super script (Invitrogen, Carlsbad, CA). qPCRs were performed in a 7900HT Fast real-time PCR system (Life technologies) using SYBR Green fuorescence with 10 ng of cDNA and quantifed using the delta–delta-cycle threshold (Ct) method(ΔΔCt). All experiments were performed in triplicate and 18S was utilized as an internal control. Mouse SIRT3 primers were purchased from Invitrogen.

Chemical cross-linking
The isolated endothelial cells were lysed with RIPA lysis buffer (containing PMSF, protease inhibitor cocktail and sodium orthovanadate, which were purchased from Santa Cruz Biotechnology) for 30 minutes at 4 °C. The lysates were centrifuged at 14,000 × *g* for 15 min at 4 °C. Then, the supernatants were treated with 2.3% glutaraldehyde at a final concentration of 5% and incubated at 37 °C for 10 minutes. Tris-HCL (50 mM, pH 7.5) was used to stop the reaction. The samples were boiled with 2× sample loading buffer at 94 °C for 5 minutes and then separated by 10% SDS-PAGE.

Western blot analysis
Protein lysates were denatured in SDS sample buffer at 100 °C for 5 min, separated on SDS-polyacrylamide gels, and blotted onto PVDF membranes (Pall Corporation, Pensacola, FL, USA) using semi-dry method. The immunoreactive bands were developed using an enhanced chemiluminescence (ECL) detection system (Pierce Biotechnology, Rockford, IL, USA) and detected using an ImageQuant LAS 400 digital biomolecular imaging system (GE Healthcare Life Sciences, Uppsala, Sweden).

***In vitro* experiment and SIRT3 transfection**
Human umbilical vein endothelial cells (HUVECs, Lonza, Basel, Switzerland) cultured in EGM medium were used in this experiment. The HUVECs cells were transfected with 100 nM of specifically designed siRNA for SIRT3 using Lipofectamine 2000 transfection reagent (Invitrogen, Carlsbad, CA, USA), according to the manufacturer's instructions. We transfected specific SIRT3 siRNA (Invitrogen, Carlsbad, CA, USA) at a 100nM concentration in the cells.

The transfected cells were treated with DCA (1mM), 2-DG (1mM), fenofibrate (1μM) and etomoxir (40 μM) for 48 hr. Glucose uptake were analyzed using the kits from Biovision Inc.
In the second set of experiments we cultured Human HK-2 cells in DMEM and Keratinocyte-SFM (1X) medium (Life Technologies Green Island NY), respectively. When the cells on the adhesion reagent reached 70% confluence, cells were cultured with conditioned media from HUVECs. The conditioned media from scramble siRNA and from SIRT3 siRNA-transfected HUVECs was collected and transferred into the HK-2 cells.

Lipid Uptake and glucose uptake
HUVECs were incubated with medium containing 0.4 μCi [^{14}C] palmitate. [^{14}C] palmitate radioactivity was measured by liquid scintillation counting. Glucose uptake assay were performed using kits from Biovision, USA.

Fatty Acid Oxidation
HUVECs were incubated with medium containing 0.75 mmol/L palmitate (conjugated to 2% fatty acid–free BSA/[^{14}C] palmitate at 0.4 μCi/mL) for 2 h. 1 mL of the culture medium was transferred to a sealable tube, the cap of which housed a Whatman filter paper disc. $^{14}CO2$ trapped in the media was then released by acidification of media using 60% perchloric acid. Radioactivity that had become adsorbed onto the filter discs was then quantified by liquid scintillation counting.

Statistical analysis
The data are expressed as the means ± s.e.m. The One way Anova Tukey test was performed to analyze significance, which was defined as $P < 0.05$, if not specifically mentioned. The post hoc tests were run only if F achieved $P < 0.05$ and there was no significant variance inhomogeneity. In each experiment, N represents the number of separate experiments (in vitro) and the number of mice (in vivo). Technical replicates were used to ensure the reliability of single values. GraphPad Prism software (Ver 5.0f) was used for the statistical analysis.

Supplemental references

Kanasaki, K., Shi, S., Kanasaki, M., He, J., Nagai, T., Nakamura, Y., Ishigaki, Y., Kitada, M., Srivastava, S.P., and Koya, D. (2014). Linagliptin-mediated DPP-4 inhibition ameliorates kidney fibrosis in streptozotocin-induced diabetic mice by inhibiting endothelial-to-mesenchymal transition in a therapeutic regimen. Diabetes *63*, 2120-2131.

Li, J., Liu, H., Srivastava, S.P., Hu, Q., Gao, R., Li, S., Kitada, M., Wu, G., Koya, D., and Kanasaki, K. (2020). Endothelial FGFR1 (Fibroblast Growth Factor Receptor 1) Deficiency Contributes Differential Fibrogenic Effects in Kidney and Heart of Diabetic Mice. Hypertension *76*, 1935-1944.

Li, J., Shi, S., Srivastava, S.P., Kitada, M., Nagai, T., Nitta, K., Kohno, M., Kanasaki, K., and Koya, D. (2017). FGFR1 is critical for the anti-endothelial mesenchymal transition effect of N-acetyl-seryl-aspartyl-lysyl-proline via induction of the MAP4K4 pathway. Cell Death Dis *8*, e2965.

Nagai, T., Kanasaki, M., Srivastava, S., Nakamura, Y., Ishigaki, Y., Kitada, M., Shi, S., Kanasaki, K., and Koya, D. (2014). N-acetyl-seryl-aspartyl-lysyl-proline Inhibits Diabetes-Associated Kidney Fibrosis and Endothelial-Mesenchymal Transition. Biomed Res Int *2014*.

Nitta, K., Shi, S., Nagai, T., Kanasaki, M., Kitada, M., Srivastava, S.P., Haneda, M., Kanasaki, K., and Koya, D. (2016). Oral Administration of N-Acetyl-seryl-aspartyl-lysyl-proline Ameliorates Kidney Disease in Both Type 1 and Type 2 Diabetic Mice via a Therapeutic Regimen. Biomed Res Int *2016*, 9172157.

Shi, S., Srivastava, S.P., Kanasaki, M., He, J., Kitada, M., Nagai, T., Nitta, K., Takagi, S., Kanasaki, K., and Koya, D. (2015). Interactions of DPP-4 and integrin beta1 influences endothelial-to-mesenchymal transition. Kidney Int *88*, 479-489.

Srivastava, S.P., Goodwin, J.E., Kanasaki, K., and Koya, D. (2020a). Inhibition of Angiotensin-Converting Enzyme Ameliorates Renal Fibrosis by Mitigating DPP-4 Level and Restoring Antifibrotic MicroRNAs. Genes (Basel) *11*.

Srivastava, S.P., Goodwin, J.E., Kanasaki, K., and Koya, D. (2020b). Metabolic reprogramming by N-acetyl-seryl-aspartyl-lysyl-proline protects against diabetic kidney disease. Br J Pharmacol.

Srivastava, S.P., Shi, S., Kanasaki, M., Nagai, T., Kitada, M., He, J., Nakamura, Y., Ishigaki, Y., Kanasaki, K., and Koya, D. (2016). Effect of Antifibrotic MicroRNAs Crosstalk on the Action of N-acetyl-seryl-aspartyl-lysyl-proline in Diabetes-related Kidney Fibrosis. Sci Rep *6*, 29884.

Sugimoto, H., Grahovac, G., Zeisberg, M., and Kalluri, R. (2007). Renal fibrosis and glomerulosclerosis in a new mouse model of diabetic nephropathy and its regression by bone morphogenic protein-7 and advanced glycation end product inhibitors. Diabetes *56*, 1825-1833.
